# Personal genome testing on physicians improves attitudes on pharmacogenomic approaches

**DOI:** 10.1371/journal.pone.0213860

**Published:** 2019-03-28

**Authors:** Kye Hwa Lee, Byung Joo Min, Ju Han Kim

**Affiliations:** 1 Center for Precision Medicine, Seoul National University Hospital, Seoul, South Korea; 2 Seoul National University Biomedical Informatics (SNUBI) and Systems Biomedical Informatics Research Center, Division of Biomedical Informatics, Seoul National University College of Medicine, Seoul, South Korea; National Chiao Tung University College of Biological Science and Technology, TAIWAN

## Abstract

In this era of clinical genomics, the accumulation of knowledge of pharmacogenomics (PGx) is rising dramatically and attempts to utilize it in clinical practice are also increasing. However, this advanced knowledge and information have not yet been sufficiently utilized in the clinical field due to various barriers including physician factors. This study was conducted to evaluate the attitudes of physicians to PGx services by providing them their own genomic data analysis report focusing on PGx. We also tried to evaluate the clinical applicability of whole exome sequencing (WES)-based functional PGx test. In total 88 physicians participated in the study from September 2015 to August 2016. Physicians who agreed to participate in the study were asked to complete a pre-test survey evaluating their knowledge of and attitude toward clinical genomics including PGx. Only those who completed the pre-test survey proceeded to WES and were provided with a personal PGx analysis report in an offline group meeting. Physicians who received these PGx reports were asked to complete a follow-up survey within two weeks. We then analyzed changes in their knowledge and attitude after reviewing their own PGx analysis results through differences in their pre-test and post-test survey responses. In total, 70 physicians (79.5%) completed the pre-test and post-test surveys and attended an off-line seminar to review their personal PGx reports. After physicians reviewed the report, their perception of and attitude towards the PGx domain and genomics significantly changed. Physician’ awareness of the likelihood of occurrence of adverse drug reactions and genetic contribution was also changed significantly. Overall, physicians were very positive about the value and potential of the PGx test but maintained a conservative stance on its actual clinical use. Results revealed that physicians’ perception and attitude to the utility of PGx testing was significantly changed after reviewing their own WES results.

## Introduction

With the development of genome analysis technology, next-generation sequencing (NGS) data is increasingly used in the clinical practice [1,2]. Cancer, rare diseases, and pharmacogenomics (PGx) are three major fields where genomics data is being applied to fulfill the promise of precision medicine [3–5]. PGx has a large impact on the clinical process because it is applicable to nearly all clinical subjects unlike patients with cancer or rare diseases [6]. It is well known that reactions to certain drugs may differ depending on the individual’s genotype. Approximately 12 CYP enzymes metabolize 70–80% of all drugs, and mutations in these genes affect drug metabolism rates [7]. When warfarin is used, the metabolism rate should be changed according to the genotype of the *VKORC1* gene as well as *CYP2C9* [8]. Carbamazepine has an odds ratio of more than 100 that causes Stevens-Johnson syndrome (SJS) and toxic epidermal necrosis (TEN) in patients with *HLA-B* * 1502 genotype [9]. Based on these studies on the relationship between adverse drug reaction (ADR) and/or responses and genotypes, the U.S. Food and Drug Administration (FDA) asked 214 drugs to indicate the relationship between genotype and drug reactivity on the drug label [10]. Additionally, several hospitals in the US have conducted pre-emptive genotyping to use PGx information properly [6,11].

However, the severity of ADRs and the importance of PGx tests to prevent them are somewhat overlooked in clinical practice. Among patients who were hospitalized because of ADRs, only 50% of them were identified their cause of admission as ADRs [12]. Even in the cases of severe ADRs, the detection rate amounted to only 95% [13]. Recent approaches to PGx still only address specific relationships between a small set of genes (or genetic variants) and a small number of drugs. There are 5,131 non-redundant proteins in DrugBank, including drug targets/enzymes/transporters/carriers, whereas only 67 drugs are listed in the U.S. FDA PGx lists [10,14]. Moreover, for the scientific utility of PGx to be translated to clinical utility, an understanding, and preference towards PGx is essential in physicians. However, most physicians are unfamiliar with genomic data and also have difficulty accepting PGx test results [15–17].

In this study, we aimed to encourage physicians to better understand genomics, ADRs, and PGx by presenting physicians with their own PGx reports using whole exome sequencing (WES). We also proposed the use of WES data as a pre-emptive test to prevent ADRs. To evaluated physicians’ changes before and after exposing of their own PGx report, we conducted a pre-test and post-test survey to confirm the understanding and attitude of physicians towards PGx, the results of their own WES analysis, and to determine changes in the knowledge and attitude of physicians. This measured physicians’ perceptions of PGx tests and the results suggest that PGx will be actively applied in the clinical setting in the near future.

## Materials and methods

### Overview of the study design and participants

**Fig 1:**
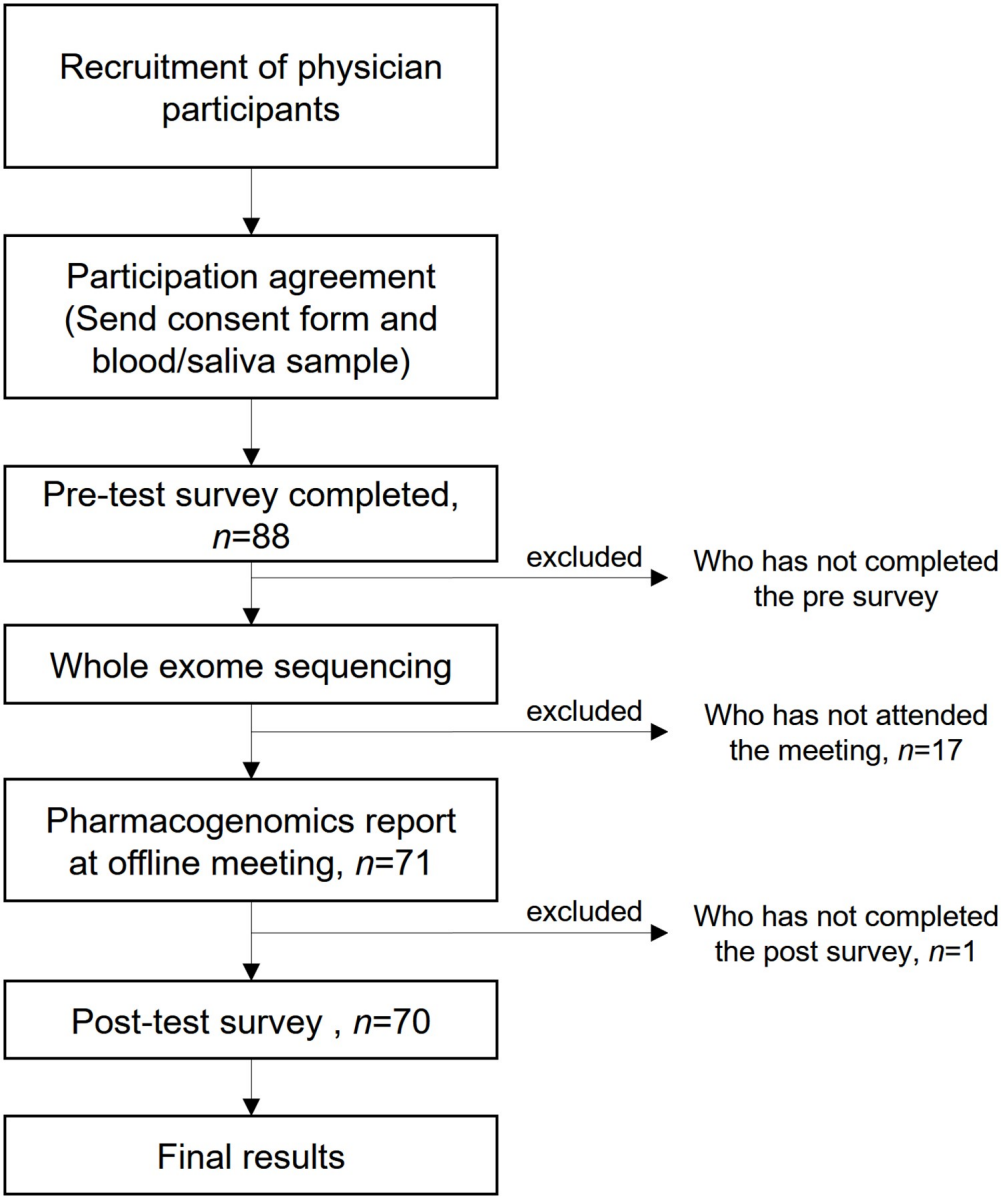
Study design and participants inclusion criteria. Of the 88 physicians who agreed to participate, there were 70 physicians who completed the entire process of participating in a pre-test/post-test survey and offline meetings to receive their PGx reports.

This study was conducted from September 2015 to August 2016. In order to recruit physicians representing various groups and specialties, we promoted this project at related conferences and seminars. Physicians who wanted to participate were asked to participate via e-mail or telephone. Once they decided to participate in the study after a telephonic consultation, they signed a participant consent form and mailed blood or saliva samples to the laboratory. Participants were selected on a first-come, first served basis to prevent research errors. The research design, as well as sequencing and analysis methods, were approved by the Institutional Review Board (IRB) of Seoul National University Hospital (Seoul, South Korea). After obtaining consent from the physicians who agreed to participate in the study and securing their blood samples were secured, we emailed the participants and administered the first survey. We performed WES for only those who completed the first survey. Once WES analysis was completed and the physicians’ personal PGx report was generated, we invited them back for an offline group meeting to explain the results. After the group meeting, participants were sent a post-test survey by e-mail. The changes in the responses between the pre-test and post-test surveys were used to measure the change of the perception and attitude toward clinical genomics and PGx, affected by the personal PGx analysis report (Fig 1).

In total, 88 physicians agreed to participate in the study, signed the consent form, completed the pre-survey, and provided blood samples. Of these, 71 participated in the PGx report meeting, and 70 completed by the second survey. In order to control the changes in basic knowledge and attitude that occurs naturally over time, we excluded participants who delayed to participate in the second survey for at least two months after participating in the personal PGx report review seminar or those who were delayed due to difficulty in coordinating the off-line meetings. Thus, the final study consisted of 70 physician subjects.

### Pre-test and post-test survey design

The study was designed in the form of a pre-test and post-test survey to evaluate the beliefs and attitudes of physicians towards clinical genomics including PGx test before and after receiving their personal PGx report. The survey consisted of eight topics, namely, basic participant information, clinical history, attitudes towards ADRs, experience with clinical genomics, experience with PGx, attitude towards PGx, an expectation of PGx testing price, and PGx interpretation report valuation. The last topic was only included in the post-test survey (Table 1). The survey was created using the Google survey, and the link for both the pre-test and post-test survey was sent to the participants via e-mail. Physicians’ e-mail or cell phone numbers were used as individual identifiers to match the pre-test and post-test survey. There were 42 questions in the pre-test survey, and 55 in the post-test survey. The questionnaires for pre-test and post-test survey were attached as Supporting Information (S1, S2 and S3 Files).

**Table 1:** Classes and items in the pre-test and post-test research questionnaires.

No	Class subject	Questionnaire items	# items
1	Basic personal information	sex, age, physician training status, working place, working place location, clinical specialty	6
2	Clinical history	Past medical history, previous history of major surgery or general anesthesia, medication history of chronic prescription, previous personal ADR* experience (chronic and ever), past week medication history, the severity of personal ADR experience	9
3	Physicians’ attitudes toward ADR	A frequency of explanation of the possibility of ADR when new prescription and reasons when not to explain, ADR estimates, genetic load for ADR occurrence, special caution drugs, most frequent ADR reported drugs,	6
4	Physicians’ experience of clinical genomics	Genetic testing prescription experience, the purpose of the genetic testing, route of knowledge for genetic testing, a degree of importance of genomics at each field: cancer, rare disease, prenatal screening, disease risk prediction, pharmacogenomics	8
5	Physicians’ experience of pharmacogenomics	Ever ordered genetic testing for drug prescription (including cancer target therapy), the specific purpose of the pharmacogenetic test order	2
6	Physicians’ attitude toward pharmacogenomics	Considering future pharmacogenomics test order, expected time to the pharmacogenomics order, barriers to applying pharmacogenomics to a clinic, ATC class of interest to apply pharmacogenomics testing, willing to change medicine according to the interpretation (patients/own family)	5
7	Physicians’ expectation of pharmacogenomics testing price	For each drug-ADR pair (warfarin-bleeding, carbamazepine-SJS/TEN, simvastatin-myopathy, clopidogrel-MI/death/stroke, valproic acid-hyperammonia), proper pricing for pharmacogenomics service using whole exome sequencing	6
8	Pharmacogenomics interpretation report valuation (post-test only)	Reliability, usefulness, convenience, willing to order to patients, proper pricing for pharmacogenomics report	5

*ADR, adverse drug reaction

### Whole exome sequencing data analysis

Genomic DNA extracted from peripheral blood cells was amplified into 175 to 250 base pair DNA fragments to collect the protein coding regions of human genome DNA using the Ion Ampliseq Exome Panel (Thermo Fisher Scientific, Waltham, MA). Library construction was performed to load the DNA samples onto the semiconductor chip by using the Ion Ampliseq Exome library kit plus covering 57,742,646 bp (1.85% of human genomic regions) as described in the manufacturer’s instructions (Thermo Fisher Scientific, Waltham, MA). Libraries were diluted to ~10 pM. Subsequently, 50 μL of the barcoded libraries were combined in sets of two barcodes. The exon-enriched DNA libraries were sequenced using the Ion Proton platform, following the manufacturer’s instructions (Thermo Fisher Scientific, Waltham, MA). Mean depth of exome sequencing ranging from 80-120x was obtained and was considered sufficient to interrogate the exons for mutations. Raw reads were initially mapped to the human reference genome build (GRCh37) using the Torrent Mapping Alignment Program (TMAP). Genome Analysis Toolkit 2.8–1 and HaplotypeCaller [18], were used to call single-nucleotide variants (SNVs) and short insertions/deletions (INDELs), respectively.

### Personal PGx report

We calculated individual drug safety score for each drug using WES data from participants who agreed to participate and completed the pre-test survey. The drug safety score for each drug was calculated based on variant deleteriousness prediction algorithm, SIFT, which having score range from 0 to 1 [19]. To calculate the drug safety score from the gene score by representing the distribution of damaging variants among the drug-related pharmacokinetic (PK)/ pharmacodynamic (PD) genes as individual gene scores, we used a previously published algorithm [20,21]. Using this algorithm, the damaging score for each gene and each drug could be estimated based on the variant score of each individual. Drug-gene pair relationship for all the PK/PD genes of the drug was extracted from DrugBank version 4.0 drug-gene interactions [14]. Of the 8,422 drug-gene interactions in total, we selected 500 most commonly prescribed drugs in the US and Republic of Korea. Among these, we finally included a total of 139 drugs that were classified into 14 system-organ-classes. These drugs were selected based on their prescription frequencies, indications and whether they have well-known PK/PD relationships (S4 File). We provided the individual drug score distribution for each indication classes, information on the variant position, alternative alleles, and SIFT score for severe damage mutations in the drug-related PK/PD genes, along with additional information on the drug metabolized by the gene when the predicted damage to the specific gene was large. To compare the distribution of individual drug scores and the distribution of drug scores in the general population, baseline data for the distribution of total ethnicity and Asian ethnicity drug scores from the 1000 genome projects was used [22].

### Personal PGx report presentation seminar

After preparation of the participants’ personal PGx report, groups of 7–10 people were gathered together for an offline group seminar. This seminar covered the structure of the report and explained how individual drug damage scores had been calculated, the function and interactions of genes associated with the drug, the inter-individual/intra-individual variability of the distribution of individual drug scores, and the interpretation of specific drugs. In addition, there were about 7–10 physician participants at each seminar, the participants could compare the results of PGx analysis with each other. There were no other materials except personal PGx report. After attending the offline meeting, participants received an e-mail with a link to the survey and were asked to complete the post-test survey within two weeks.

### Statistical analysis

In order to identify changes in the attitudes and perceptions towards PGx in physicians who received personal genome-based PGx reports, we measured changes in their responses to the same items in the pre-test and post-test surveys. The post-test survey included additional items for feedback on the content and composition of the report. To compare the pre-test and post-test survey results, we converted ordinal variables to scores and performed a Wilcoxon signed rank test. Categorical variables including the binary variables ("yes" or "no" questions) were subjected to a McNemar test. The level of statistical significance was determined at *p* value <0.05. The statistical analysis was supported by the Medical Research Collaborating Center (MRCC) in Seoul National University Hospital. All statistical analyses were conducted using the R statistical package (ver. 3.5.1) [23].

## Results

### Physician-participants basic characteristics

**Fig 2:**
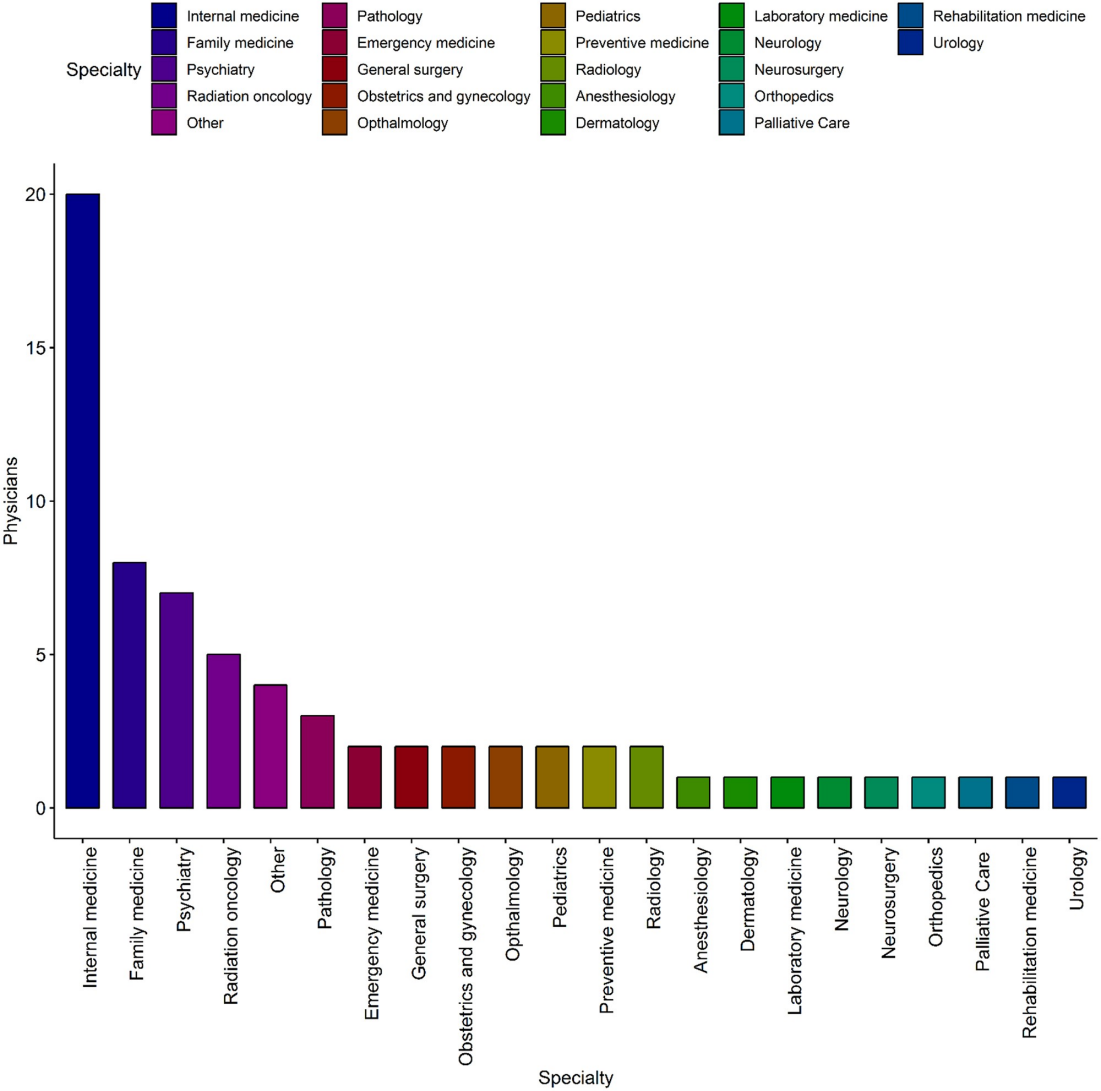
Specialty distribution of 70 participating physicians. Most of the 70 physicians participating in the study belonged to 22 specialties. The other cases included were not official specialists.

The final study group of subjects comprised a total of 70 physicians who completed a pre-test survey, attended an offline meeting, reviewed the results of their personal PGx report, and completed a post-test survey. Their basic characteristic was represented in Table 2. The group of participants included 38 males and 32 females, and their ages ranged mostly from 30 to 50 years. Of these, 31 (44.3%) were at the fellowship stage after specialty board, and 17 (24.3%) were professors at university hospitals; 59 (87%) were working at medical schools and tertiary hospitals. Most of the physicians practiced in the metropolitan Seoul area (82.4%). A wide variety of physicians from 22 specialties participated in the present study (Fig 2).

**Table 2:** Basic characteristics of physician participants.

Items		Number (%)
Sex	Male	38 (54.29)
	Female	32 (45.71)
Age	30~39	33 (44.57)
	40~49	30 (42.86)
	50~59	6 (8.57)
	60≤	1 (1.43)
Level of training	Internship	6 (8.57)
	Specialty	15 (21.43)
	Fellowship	31 (44.29)
	Professor	17 (24.29)
	Other	1 (8.57)
Working place	Medical college	15 (21.43)
	Tertiary hospital	44 (65.86)
	Secondary hospital	2 (2.86)
	Clinic	4 (5.71)
	Research lab	1 (1.43)
	Company	1 (1.43)
	Other	3 (4.29)
Working area	Metropolitan area including Seoul, Incheon, and Gyeonggi province	57 (82.43)
	Other	13 (18.58)
Specialty	Internal medicine	20 (28.57)
	Family medicine	8 (11.43)
	Psychiatry	7 (10.00)
	Radiation oncology	5 (7.14)
	Pathology	3 (4.29)
	Emergency medicine	2 (2.86)
	General surgery	2 (2.86)
	Radiology	2 (2.86)
	Pediatrics	2 (2.86)
	Obstetrics and gynecology	2 (2.86)
	Opthalmology	2 (2.86)
	Preventive medicine	2 (2.86)
	Dermatology	1 (1.43)
	ENT	1 (1.43)
	Anesthesiology	1 (1.43)
	Laboratory medicine	1 (1.43)
	Orthopedics	1 (1.43)
	Rehabilitation medicine	1 (1.43)
	Neurology	1 (1.43)
	Neurosurgery	1 (1.43)
	Urology	1 (1.43)
	Other	4 (5.71)

### Clinical history of participants

Thirty-five patients (50%) out of seventy responded that they do not have any medical history or chronic disease. The remaining 35 respondents said that they have one or more of the following: cancer (5), allergy (9), pulmonary tuberculosis and latent tuberculosis (5), autoimmune disease (2), diabetes (2), hyperlipidemia (3), fatty liver / hepatitis / cirrhosis (2), thyroid disease (2), and other diseases (8 patients). Sixteen participants (22.9%) had undergone major surgery with general anesthesia. Of all the participants, 14 (20.0%) were currently taking chronic medication, and among these, three patients (21.4%) had experienced ADRs to chronic medication.

### Physicians’ attitude toward ADR

In order to investigate the physicians’ change of awareness and attitude regarding ADRs after reviewing their individual PGx report, we asked four questions in the pre-test and post-test surveys: 1) How often do you explain the potential for ADR when you prescribe a new medication to your patients? 2) Why not explain the possibility of ADRs? 3) Of the patients you prescribed, how many patients did you think would have experienced ADRs? 4) How much do you think, will genetic makeup contribute to the occurrence of ADRs?

**Fig 3:**
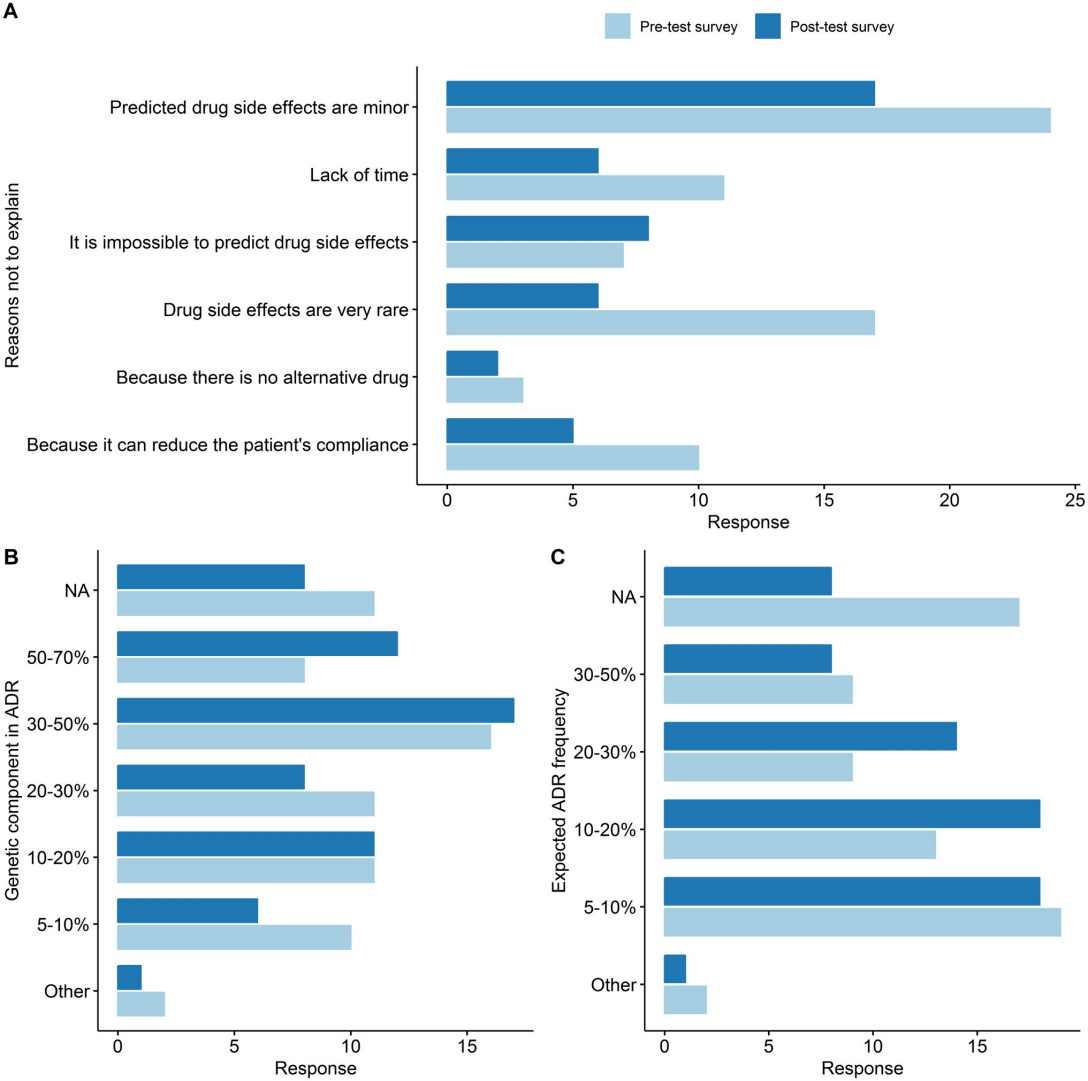
Changes in physicians’ belief regarding ADRs. (A) The reasons why physicians did not explain the possibility of ADRs to their patients when they prescribe new drugs, the most commonly chosen answer was “Because the expected ADR of the drug was mild.” in both pre-test and post-test survey. The ranking was changed for the other remaining answers. (B) The investigated physicians’ perception of genetic predisposition contributing to ADRs. (C) represents the physicians’ perception of the patients who experienced side effects from prescribed drugs. Pre-test survey responses are represented in light blue and post-test survey responses are represented in deep blue color.

In their pre-test surveys, 20 (28.6%) and 43 physicians (61.3%) selected the "Always explain" and "Sometimes explain" option, respectively, when indicating whether they would explain the potential side effects of giving new medication to a patient. These showed a statistical difference at the post-test survey with 26 (37.1%) and 42 (60.0%) participants but not significant (*p*-value = 0.07, McNemar’s test). When physicians chose the answers "do not explain" or "explain occasionally", we asked another question to evaluate the reasons not to explain. Forty-seven and forty-six physicians answers for the further question, respective. In this question, which allowed multiple choices, they stated that this was mainly because they expected the ADRs to be mild, 55.8 (24 out of 43) and 40.5% (17 out of 42) in the pre-test and post-test surveys, respectively. However, there was a fluctuation in the remaining rankings, “Because of the rarity of ADRs” was ranked from second to third and “Because of the unpredictability of ADRs” was ranked from fifth to second in the pre-test and post-test surveys, respectively (Fig 3, S1 Table). Next, the physicians’ expectation of the effect size of individual genomic makeup on the occurrence of ADRs was assessed on seven scales and were scored. The physicians’ expectation of the degree of contribution of the genetic component to ADR occurrence was significantly increased after review of their own PGx report. The mean score in the pre-test survey was 3.47 (± 1.78) and in the post-test survey 4.24 (± 1.89) (*p* value <0.0023) (Fig 3 and Table 3). The answer to the question of "How many of your patients, do you think, have experienced drug side effects?" was scored as 1 point for less than 5% and 6 points for more than 50%. Interestingly, the belief in the likelihood of developing ADR has also changed, the mean score was increased in the post-test survey (2.85 ± 1.30) than in the pre-test survey (2.57 ± 1.39), which was statistically significant (p value <0.014) (Fig 3 and Table 3).

**Table 3:** Changes in knowledge and attitude of physicians of the genetic components of ADRs and expected ADR frequency.

	Pre-test questionnaire Score		Post-test questionnaire Score		Diff (Post-test questionnaire—Pre-test questionnaire)		*p* value^a^
	n	mean(SD)	n	mean(SD)	n	mean(SD)	
The genetic component in ADRs	70	3.47 (1.78)	70	4.24 (1.89)	70	0.77 (2.1)	0.0023
Expected ADR frequency	70	2.59 (1.46)	70	3.03 (1.38)	70	0.44 (1.45)	0.014

^a^ Wilcoxon rank sum test

### Physicians’ experience of clinical genomics

We used the following four items to evaluate the genetic test prescribing experience of physicians: 1) Have you ever prescribed a genetic test? 2) If you have had prescription experience with genetic tests, what was the purpose? 3) Where did you get the knowledge and information about the genetic screening prescription? 4) How do you think the genetic variation contributes to each of the representative fields to which genetic testing is applied?

**Fig 4:**
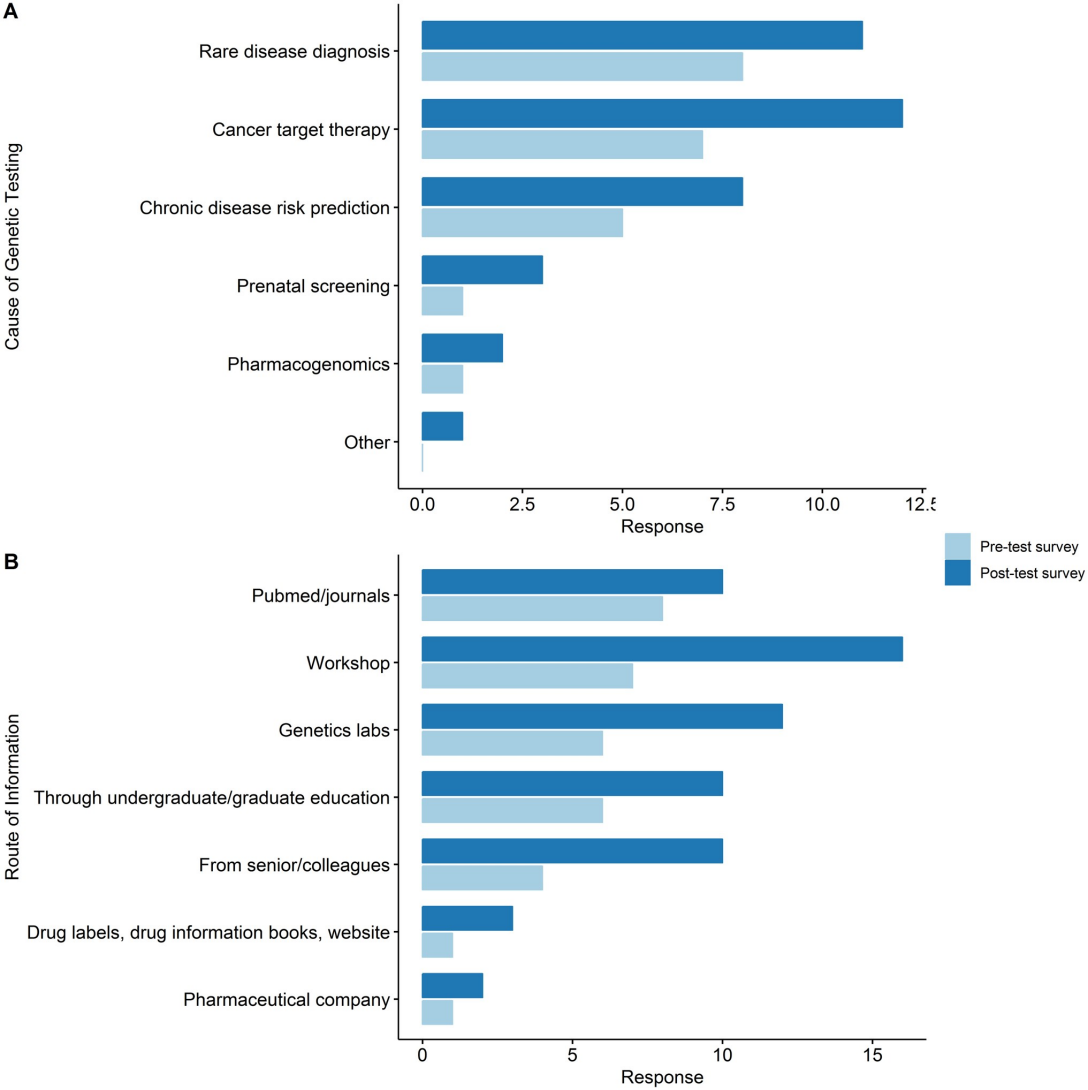
Changes between the pre-test and post-test survey in physicians’ attitude and expectations towards clinical genomics. (A) The reasons for which the physicians’ prescribed genetic testing. (B) The sources of information used by the physicians when they prescribed a genetic test.

Of the total 70 participating physicians, 18 (25.7%) and 22 (31.4%) did have an experience in prescribing genetic testing in the pre-test and post-test survey respectively. Among 18 who have had experience of genetic testing, three have changed the answer to “I don’t have any experience of genetic testing.” at the post-test survey. At post-test survey, there were seven more physicians who have had experience in genetic testing. Independently from the question of previous genetic testing prescription experience, for the question of the reason that they have had prescribe genetic testing, 18 and 25 physicians answered, respectively. In the pre-test survey, the rank of diseases for genetic testing were as follows: Rare disease diagnosis (38.1%, *n* = 8) → Cancer target therapy (38.3%, *n* = 7) → Chronic disease risk prediction (10.6%, *n* = 5) → Prenatal screening (2.1%, *n* = 1) and Pharmacogenomics (2.1%, *n* = 1). On the other hand, the rankings in the post-test survey were changed as follows: Cancer target therapy (54.54%, *n* = 12) → Rare disease diagnosis (50%, *n* = 11) → Chronic disease risk prediction (36.4%, *n* = 8) → Pre-natal screening (13.6%, *n* = 3) → Pharmacogenomics (9.1%, *n* = 2) (Fig 4). There has also been a growing body of the response to obtaining the knowledge and information needed to prescribe genetic testing from 33 cases in the pre-test survey to 63 cases in the post-test survey. As a source of information, journals were the first (24.2%) in the pre-test survey, whereas in the post-test survey, the academic workshops and seminars were at the top (25.4%) (Fig 4).

Finally, we examined the physicians’ evaluation of the importance of genomes in five areas where genetic testing is applied (cancer target therapy, rare disease diagnosis, prenatal diagnosis, disease risk prediction, PGx). Significance was assessed by five Likert scales: Because there were a few answers chosen "not important" or "less important" for all five areas, we combined "less important" and "not important" answers together and made these responses to four Likert scales in the analysis process. Table 4 shows the scores of the pre-test and post-test survey by converting the Likert scale to score (4 to very important and 1 to not important). There were statistically significant increases in the perception of the importance of genomes in three areas: Cancer target therapy, rare disease diagnosis and pharmacogenomics.

**Table 4:** Changes in perception of physicians of the expected contribution of genetic component for each domain of cancer, rare disease, prenatal screening, disease risk prediction and PGx at pre-test and post-test questionnaire.

Clinical Fields	Pre-test questionnaire Score	Post-test questionnaire Score	Diff (Post-test questionnaire—Pre-test questionnaire)		*p* value*
	mean (SD)	mean (SD)	n	mean (SD)	
Cancer target therapy	4.35 (0.78)	4.57. (0.67)	69	0.26 (0.83)	0.01
Rare disease diagnosis	4.43 (0.83)	4.66. (0.63)	70	0.23 (0.92)	0.0287
Prenatal screening	4.16 (0.96)	4.34 (0.8)	69	0.22 (0.95)	0.0852
Chronic disease risk prediction	3.61 (1.04)	3.79 (1.03)	70	0.17 (1.04)	0.1583
PGx	4.1 (0.94)	4.33 (0.81)	69	0.26 (0.83)	0.0104

* Wilcoxon rank sum test

### Physicians’ experience, attitude and expectation toward PGx test

In order to identify the changes in PGx prescription experience and the attitudes of physicians involved, we designed the following five questions: 1) Have you ever prescribed a PGx test? 2) If you have prescribed a PGx test to your patient, what was its purpose? 3) To what extent do you consider the use of PGx testing in future clinical studies and research? 4) If you plan to use the PGx test for medical practice and research, at what point do you expect it to be available? 5) What are the biggest obstacles to the use of PGx in clinical practice and research?

**Fig 5:**
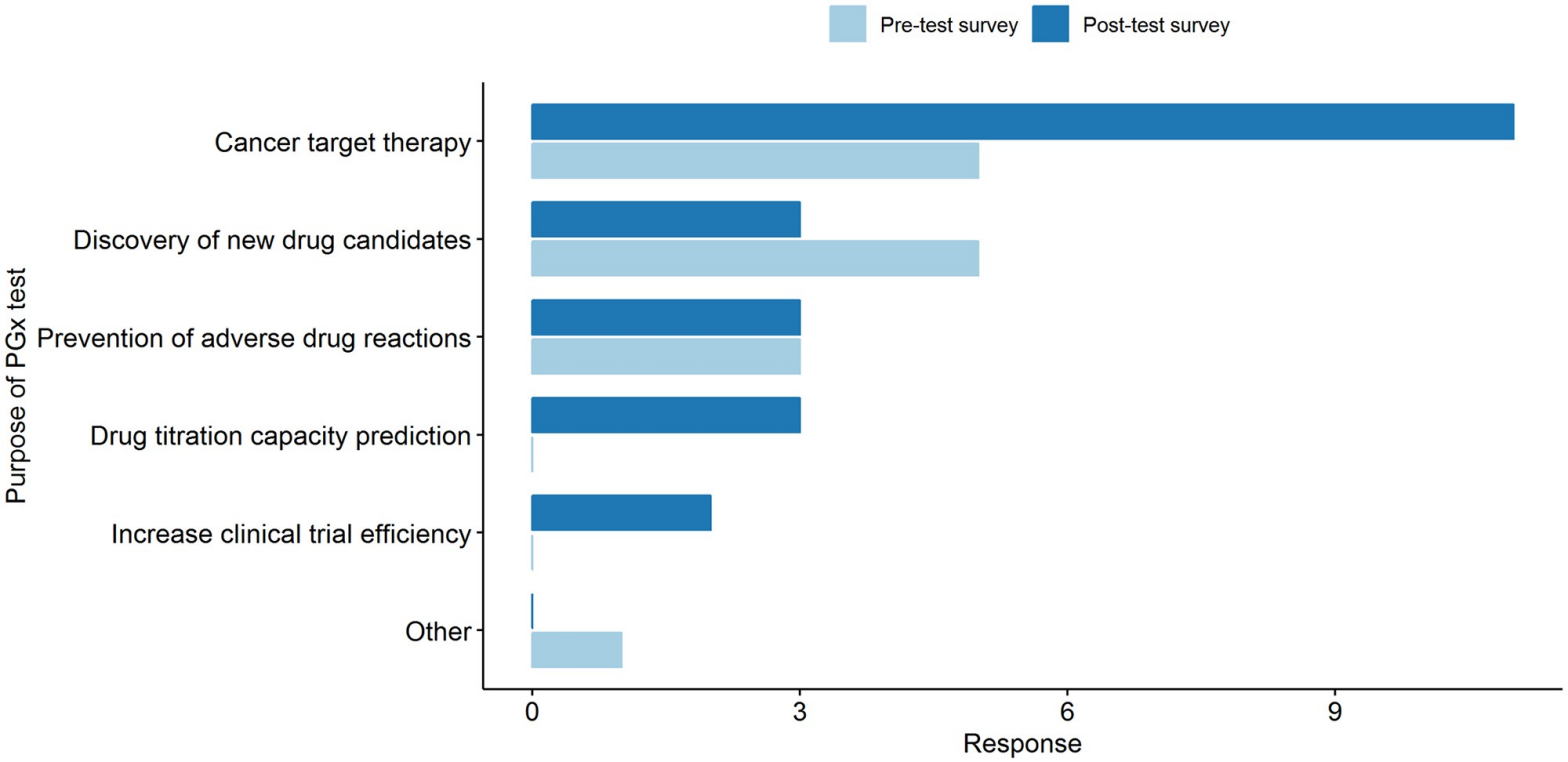
Physicians’ awareness of the importance of genomic data in five key areas. Each area shows cancer diagnosis and chemotherapy, chronic disease risk prediction, pharmacogenomics, prenatal diagnosis and diagnosis and treatment of rare diseases from top left to bottom right.

There was no significant difference in the number of physicians who had any experience with PGx test prescription, with 10 and 11 in the pre-test and post-test survey, respectively (p value = 1). The reason for prescribing the PGx test which collected from the question allowed multiple choices, the first ranked one in both the pre-test and post-test survey was “for cancer target therapy”, with five cases (35.7%) in the pre-test survey, and 11 cases (50.0%) in the post-test survey. Although there was no difference in the number of physicians who had experience of PGx testing, the cause of prescription of the genetic tests became much diverse. As represented in Fig 5, the diversity of the prescription increased in the post-test survey and there were three answers in the post-survey, “To find new drug candidates” which was zero in the pre-test survey. Likewise, there were two cases in the post-test survey alone stating “To increase the clinical trial efficiency” as the reason for prescription (Fig 5).

**Fig 6:**
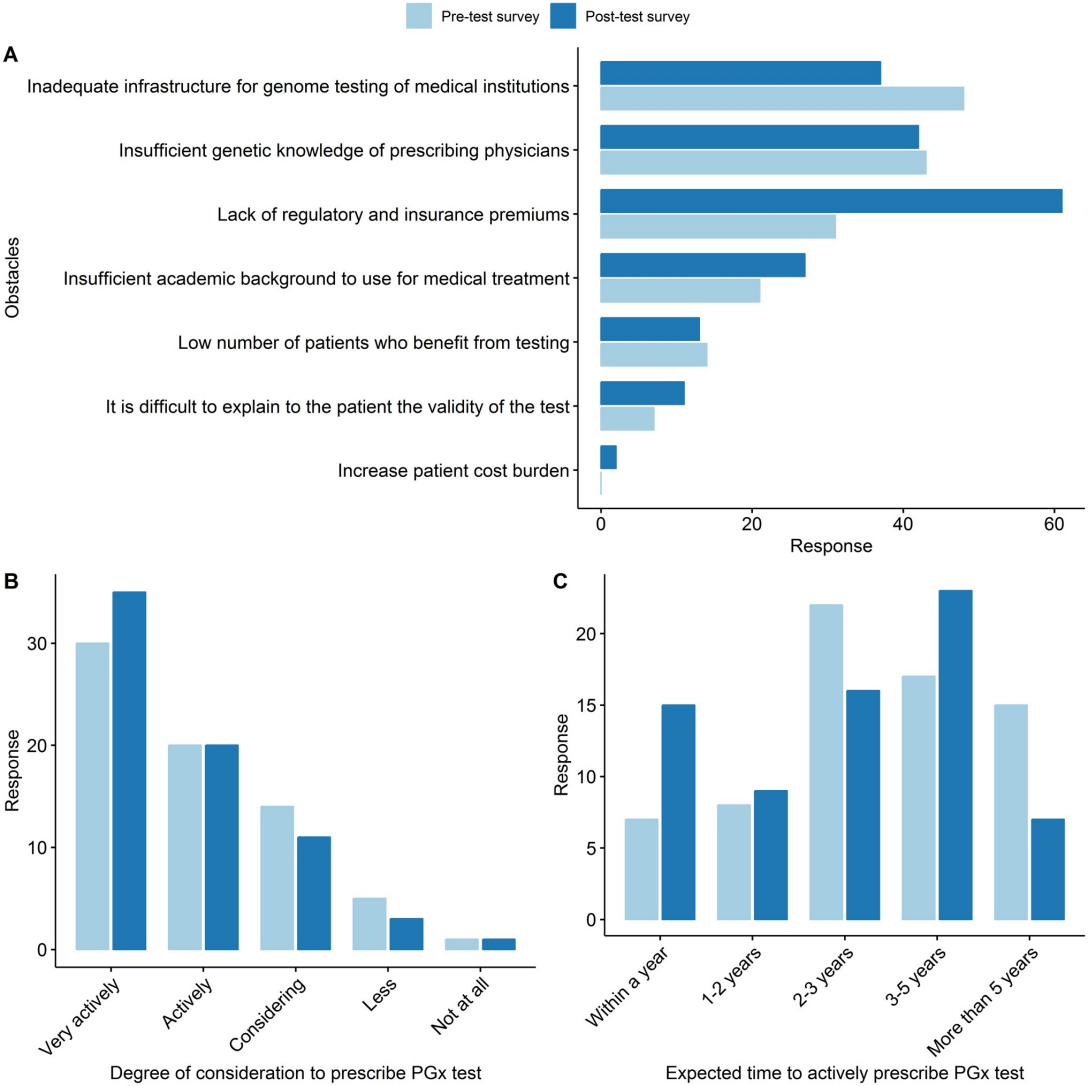
Physicians’ attitude and expectations of pharmacogenomics testing. (A) The obstacles that physicians perceive in prescribing the PGx test. In the pre-test survey, ‘Inadequate infrastructure for genomic testing of medical institutions’ is the top obstacle, whereas ‘Lack of regulatory and insurance premiums’ is the top in post-test survey. (B) The responses to how physicians are considering PGx testing in the future. The ‘very actively considering’ response increased significantly from 30 in the pre-test survey to 35 in the post-test survey. (C) The changes in response to when physicians think that the PGx test will be actively used in the future.

For the question about obstacles to prescribe PGx, although there were some differences in the ranking of the top three remained the same in the pre-test survey and the post-test survey: lack of genomic knowledge in prescribing physicians, insufficient benefits such as insurance benefits and inadequate infrastructure for the genome testing of each medical institution (Fig 6). When asked about their plans to use PGx tests in future clinical or research setting, the answer "very actively considered" was significantly increased from 30 (42.8%) in the pre-test survey to 35 (50.0%) in the post-test survey (Fig 6). Interestingly, when asked about the time when the PGx test is expected to be used clinically in the pre-test survey, the choices were ordered as: the next 2–3 years (22, 31.5%) → between 3–5 years (17, 24.3%) → after 5 years (7, 10.0%) but in post- survey, rankings were significantly different in the next 3–5 years (23, 32.9%) → between 2–3 years (16, 22.9%) → within 1 year (7, 10.0%) (Fig 6).

### Valuation of PGx information

This section is designed to determine how physicians value PGx testing in current health care systems. In order to evaluate the test value, we asked physicians about the expected cost of genetic testing to diagnose the commonly used drug-ADR pairs for which serious side effects and causative genotypes have been identified in the US FDA table of PGx [10] and the Clinical Pharmacogenetics Implementation Consortium (CPIC) guidelines [24]. The drug-ADR pairs were as follows: 1) Warfarin-Bleeding, 2) Carbamazepine-SJS/TEN, 3) Simvastatin-Myopathy, 4) Clopidogrel-Myocardial Infarction/Death, and 5) Valproic acid-Hyperammonemia. This question was made on the basis of the Korean currency, which matched the usual price of the service in Korean medical systems. The price was converted to US dollars for this manuscript. In the pre-test and post-test surveys, the most frequently chosen prices ranged from 9$ to 90$. The expected cost of the five drug-side-effects pairs was higher in the post-test survey than in the pre-test survey except for Carbamazepine-SJS and TEN, but this trend showed no statistically significant difference. Also there was no statistical difference for the expected cost of PGX test using WES in the pre-test and post-test survey but the rankings were different, so the most frequently chosen answers in the pre-test survey were between 250$–450$, and those in the post-test survey were between 90$-250$. (S1 and S2 Figs).

## Discussion

In this study, we measured the changes in personal knowledge and attitudes of physicians after receiving their own personalized PGx reports using WES data. Overall, physicians’ perception of ADR occurrence and the contribution of personal genomic variability to ADRs changed dramatically. The physicians’ perceived prevalence of ADRs was increased significantly in the post-test survey compared to pre-test (Table 3) As the introduction of genomic tests in clinical practice has been very rapid over a period of about one year, physicians’ genetic test prescription patterns and getting information resources have been diversified. However, institutional support such as NGS testing infrastructure, insurance benefits, and concerns about the lack of knowledge in medical personnel are still considered major barriers in the use of genetic testing in clinical practice, suggesting that systematic support is needed to accelerate acceptance of PGx testing in clinical practice.

Interestingly, to review their own PGx test report aroused physicians’ attention to ADRs. It is well known that physicians tend to overlook ADRs. According to the previous study, more than half of the hospitalizations due to the pre-existing ADRs were not recognized by physicians [12]. Most physicians prescribe drugs empirically focused on effect [25]. This empirical prescription pattern could not take account of the patient’s genomic diversity. The physicians participating in this study has been changed of their attitude related to PGx as well as awareness of ADRs after receiving their own PGx testing. It suggests that physicians who gained more insight into the inter-individual variability of PGx became to have more attention to ADRs which could be driven by the same variability. Therefore, the education of PGx testing will not only improve physicians’ knowledge of genomics, but also might have educational effects on the neglected but preventable ADRs.

During the study period, the number of physicians with experience in prescribing genetic tests increased, the number of target diseases varied, and the source of knowledge to acquire genetic testing information also have been diversified (Fig 4). The most common cause of prescription was a rare disease diagnosis and cancer target therapy in both pre-test and post-test survey, but the prescription cases increased in chronic disease prevention, prenatal screening and PGx as well. There was no significant difference in the number of physicians with PGx prescribing experience, but prescription indications have been diversified and new use purpose appeared in the post-test survey such as the development of new drugs and to improve the efficiency of clinical trial (Fig 5). The physicians who answered that they very actively consider PGx testing increased from 42.8% in the pre-test to 50% in the post-test survey. However, the top chosen answer for the question of the expected timing of actualization of PGx service in clinical practice was “within 2–3 years” (31.5%) in the pre-test and “within 3–5 year” (32.9%) in the post-test survey. This suggests that physicians felt that there are more to consider in order to prescribe PGx test in clinics after they identified the realities of the service that had been known vaguely. In this regard, the fact that the top chose answer was “Lack of regulatory and insurance premiums” for the question of “What are the biggest obstacles physicians think for the clinical introduction of PGx services?” suggest there should be a sophisticated systemic support such as insurance and regulation rules for the successful introduction of genomic testing including PGx.

Physicians are accustomed to evidence-based practice (EBM) based on traditional randomized controlled trials (RCTs). However, we could hardly identify various and unexpected ADRs of drugs through RCTs because patients involved in RCTs usually have homozygous and better clinical characteristics then real world patients. In RCTs we could only able to investigate the efficacy of drugs [26]. In addition, more than 75% of prescription drugs are metabolized by the CYP family [27] and 25% of outpatients are prescribed drugs with PGx information attached to the drug label [28]. As such, most patients are currently receiving PGx-related drugs, but PGx tests are not actively being used. Therefore, it is one of the major implications of this study that physicians to take PGx test of themselves and to see their own genomic profiles promote them to understand general genomics including PGx and to remind ADRs.

To our knowledge, this study was the first to examine physicians’ perception of the application of clinical genomics focused on PGx by using their own PGx analysis through WES, but it was consistent with previous studies. Physicians’ biggest obstacles to clinical use of genetic testing are lack of knowledge/experience and self-confidence [16,29]. These were important obstacles in our research as well. The physicians who participated in this study mostly included tertiary medical specialists from various fields, all of whom were interested in genomics and were recruited from relevant seminars. Their actual experience with genetic testing was around 15%. Still, the lack of knowledge of physicians was one of the main obstacles to the introduction of PGx. This is due to the fact that the speed of new knowledge discovery in the field of genomics overtakes the pace of existing medical education. Physicians showed a very positive attitude towards the clinical use of PGx testing, but they were relatively cautious about the time and cost to patient care which is consistent with previous studies [29,30]. Therefore, for the clinical genomics testing, continuous efforts should be made for new knowledge education for physicians.

The potential source of bias for the study is that the influence of the seminar could not be excluded. The contents of the seminar only focused on the genomic variability and principle of PGx, but still the physicians could be affected not only their own genomic profile but also a perception of other participants in the same seminar. In addition, for the PGx report, this study used not the existing PGx SNPs but previously published in-house algorithm. So the physicians’ acceptance and understanding of the contents of PGx report might be relatively low. It could not completely rule out the possibility of this method affecting the post-test survey response. The other limitation is the major body of physicians in this study were from the metropolitan area. Because the physicians in the rural area are under pressure to develop more diverse and advanced services, so physicians in the rural area might have a more conservative attitude than physicians participated in our study. The answers to the questions of the appropriate cost of the test to predict the drug-side effect pairs in the study would not be applicable to other countries having different economic and health insurance system.

A preemptive approach is very useful for PGx test because physicians do not know when drugs will be exposed to each patient [31,32]. In order to bring this pre-emptive approach into practice, physicians need to take an active attitude with insight into PGx and ADRs. In previous studies, it has been reported that the education using the patient’s sequencing data was very effective for medical students [33]. In order for the scientific utility of PGx information to be reflected in actual clinical practice, there are a wide variety of barriers to overcome including sufficient knowledge of genomics among physicians, changes in the clinical process, consideration of race differences in PGx and confirming cost-effectiveness of the tests. Of these, the most proactively required is to improve the physician’s knowledge of genomics through pre-education and drawing their commitment to personalized medicine. Interestingly, what we provided to physicians in our study was only a PGx analysis results, the expectation of genetic contribution to the other genetic areas also showed an increase (Table 4). This study has shown that approaches including physicians’ own genome data analyses can lead to very interesting results and lead physicians to change their attitudes and improve overall knowledge of genomics.

### Conclusion

This study identified that the attitudes and perceptions of the entire clinical genomics including PGx of physicians show significant changes before and after their own WES based PGx reports.

## Supporting information

S1 Fig This figure shows the physicians’ estimation of appropriate patient’s cost for PGx testing to predict each drug-side effect pair. (TIF)

S2 Fig This figure shows that the physicians’ estimation of the appropriate cost for clinical application of PGx testing using whole exome sequencing. (TIF)

S1 Table Physicians answers for the reasons not to explain the possibility of ADRs to their patients. (DOCX)

S1 File Pre-test and Post-test survey questionnaires in English. (PDF)

S2 File Pre-test questionnaire in Korean. (PDF)

S3 File Post-test questionnaire in Korean. (PDF)

S4 File List of drugs used to make pharmacogenomics report. (CSV)
