# Supplementary figures and images for: Personal genome testing on physicians improves attitudes on pharmacogenomic approaches
Source: PLoS One. 2019 Mar 28;14(3):e0213860. doi: 10.1371/journal.pone.0213860 (PMC6438681; doi:10.1371/journal.pone.0213860)

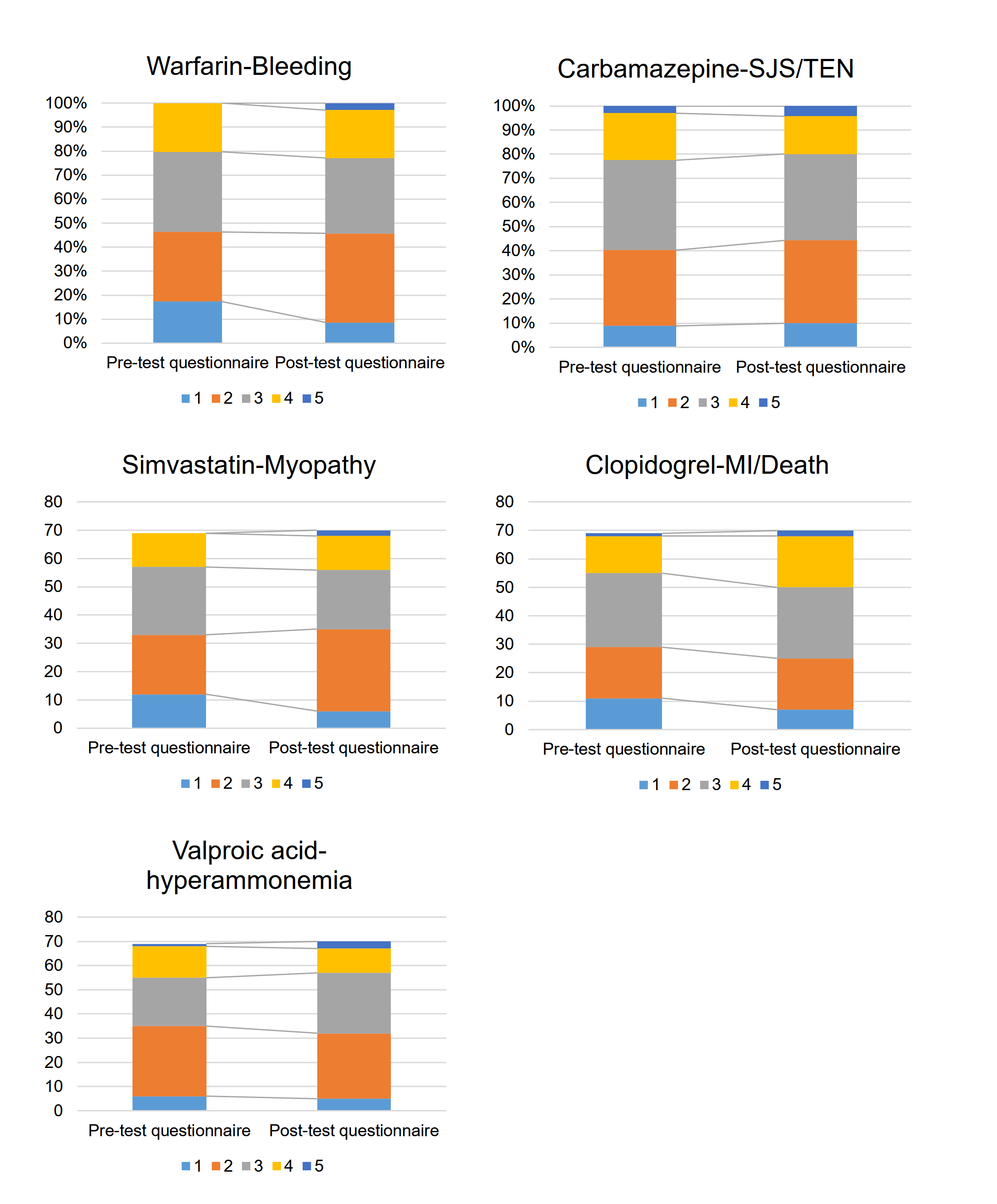

Supplement: S1 Fig — (TIF) [file pone.0213860.s001.tif]

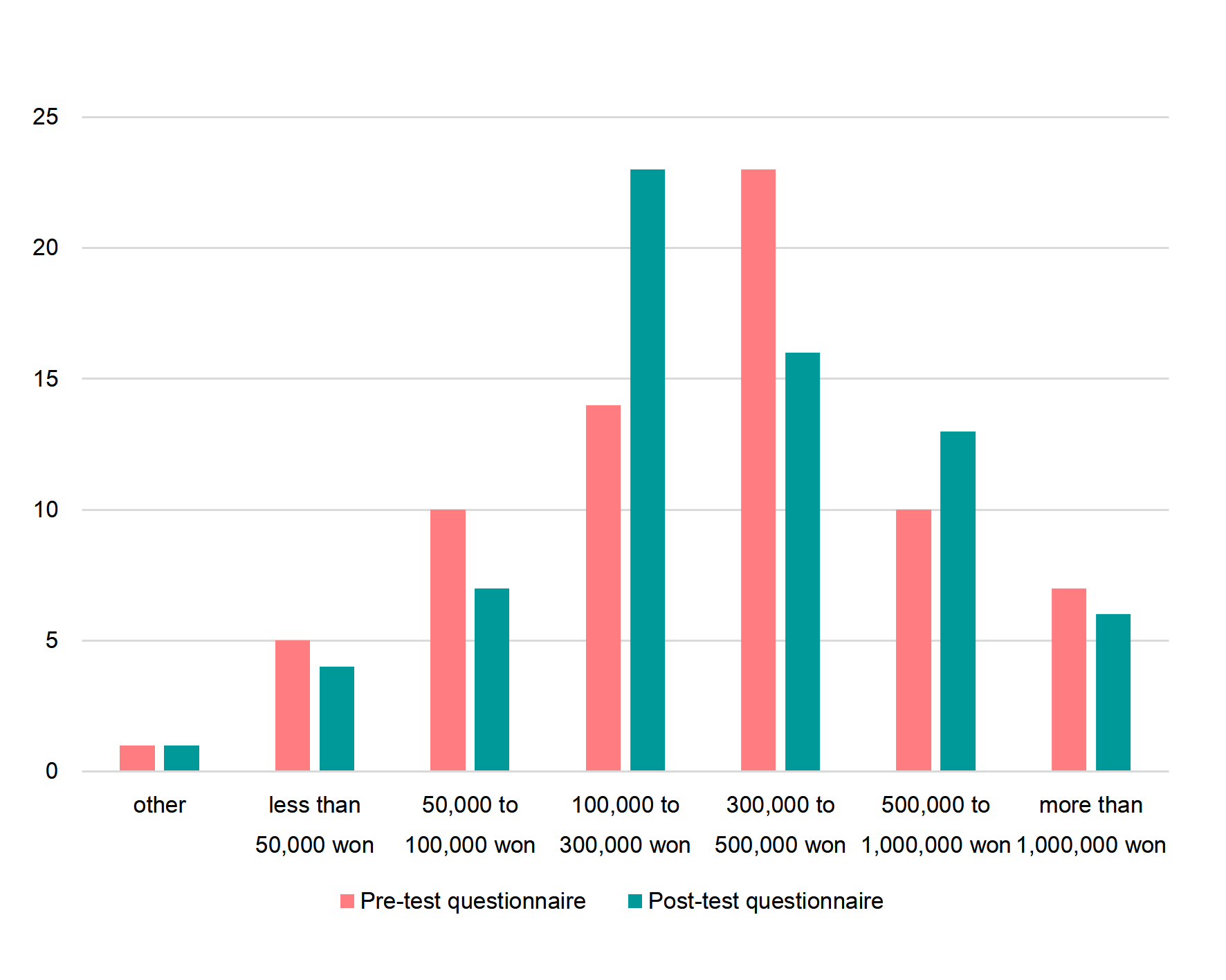

Supplement: S2 Fig — (TIF) [file pone.0213860.s002.tif]
