# Supplementary material for: Personal genome testing on physicians improves attitudes on pharmacogenomic approaches
Source: PLoS One. 2019 Mar 28;14(3):e0213860. doi: 10.1371/journal.pone.0213860 (PMC6438681; doi:10.1371/journal.pone.0213860)
Supplement: S1 Table — (DOCX) [file pone.0213860.s003.docx]

**Supplementary Table 1. Physicians answers for the reasons not to explain the possibility of ADRs to their patients.**

| Reasons not to explain | Pre-test survey response^a^ (%) | Post-test survey response^b^ (%) |
| --- | --- | --- |
| Predicted drug side effects are minor | 24 (55.8) | 17 (40.5) |
| Drug side effects are very rare | 17 (39.5) | 6 (14.3) |
| Lack of time | 11 (25.6) | 6 (14.3) |
| Because it can reduce the patient's compliance | 10 (23.3) | 5 (11.9) |
| It is impossible to predict drug side effects | 7 (16.3) | 8 (19.1) |
| Because there is no alternative drug | 3 (7.0) | 2 (4.8) |

^a^Total number of physicians answered for this question were 43 at pre-test survey. ^b^Total number of physicians answered for this question were 42 at post-test survey.
