# Supplementary material for: Personal genome testing on physicians improves attitudes on pharmacogenomic approaches
Source: PLoS One. 2019 Mar 28;14(3):e0213860. doi: 10.1371/journal.pone.0213860 (PMC6438681; doi:10.1371/journal.pone.0213860)
Supplement: S1 File — (PDF) [file pone.0213860.s004.pdf]

DoctorSeq Project: questionnaire

This questionnaire was designed to identify physicians' perceptions of changes before and after receiving their own pharmacogenomic testing. Before this questionnaire, blood submitted with your consent has undergone a whole exome sequencing. Using this data, we conducted pharmacogenomics (PGx) tests. Once you have completed this questionnaire, we will provide you with a report on drug safety predictions through analysis of mutations in your pharmagogenomics profile. If you participate in the second questionnaire about two weeks after you receive the PGx report, we will check your perception changes before and after receiving the report.

This questionnaire takes 35 minutes and takes about 20 minutes. All your responses will be anonymous.

Identity Matching Procedures for Pre-Post Survey Comparisons

1. Identity matching work is required to track changes in pre-post survey results. Please enter your cell phone number. This number is not used except for form matching. *

   ____

   Basic Information

2. What is your gender? *

   1) Male 2) female

3. What is your age range?

   1) 20's 2) 30-39 years 3) 40-49 years 4) 50-59 years 5) 60 years or older

4. What level of training is your?

   1) Graduated from medical school
   2) Internship completion
   3) Specialist
   4) Clinical instructor or above fellowship

5) Associated professor or higher
6) Other

5. What is your current workplace?
    1) University or medical school
    2) 3th level of general hospital or university hospital
    3) Secondary hospital (30-99 beds)
    4) Clinic or private hospital
    5) Laboratory
    6) Company
    7) Other
6. Where is your current working area?
    1) Seoul
    2) Gyeonggi province
    3) Gangwon province
    4) Chungsheong province
    5) Kyungsang province
    6) Jeolla province
    7) Jeju island
    8) Other
7. What is your specialty?

    ____

General health survey

8. Please record any illnesses that you are currently suffering or have previously been diagnosed with.

1) Stroke
2) Heart disease such as angina
3) Hypertension
4) Hyperlipidemia
5) Diabetes
6) Tuberculosis
7) Chronic bronchitis and lung diseases
8) Cancer
9) Autoimmune disease
10) Allergy
11) Liver disease
12) Thyroid disease
13) Bone disease
14) None
15) Other

9. Have you under gone surgery with general anesthesia?

1) Yes 2) No

Survey on the experience of drug side effects

10. Do you currently take one or more medications daily for three or more months?

1) Yes 2) No

11. If you have a prescription drug that you take steadily over three months, have you experienced any side effects while taking the medicine?

1) Yes 2) No 3) I don't have any medicine to take steadily

12. Have you taken any medication for the past week? (Including prescription drugs and generic drugs)

1) Yes 2) No

13. If you have taken your medication even once during the past week, please write down the name of the medicine as you remember it.

____

14. Have you ever experienced side effects of drugs once?

1) Yes 2) No 3) I have never taken medicine

15. If you have ever experienced a side effect, what was the side effect?

	1	2	3	4	5	
Temporary and light						Very serious and need to be changed the medicine immediately

16. If you have ever experienced a side effect of the drug, please write down the drug and its side effects. (E.g., Ibuprofen-Heartburn, Augmentin-urticaria ..)

____

Survey on anticipated side effects of prescription drugs

17. How often do you explain the potential for ADR when you prescribe a new medication to a patient?

	1	2	3	4	
Almost never					Always

18. Why not explain the possibility of ADR? (Check all that apply.)

1) Predicted drug side effects are minor

2) Because the possibility of adverse drug reactions is very low
3) It is impossible to predict drug side effects
4) Because there is no alternative drug even if side effects occur
5) Patients can reduce drug compliance
6) There is a shortage of time to explain due to the tight time
7) Other:

19. Of the patients you prescribed, how many patients did you think would have experienced ADR?
    1) Less than 5%
    2) 5 to 10%
    3) 10 to 20%
    4) 20 to 30%
    5) 30 to 50%
    6) More than 50%
20. How much do you think genetic factors will contribute to ADR?

1) Less than 5%
2) 5 to 10%
3) 10 to 20%
4) 20 to 30%
5) 30 to 50%
6) 50 to 70%
7) More than 70%
8) Other:

21. When prescribing medication to a patient, do you have any medications that you think you need to be especially cautious about adverse drug reactions?
    1) Yes 2) No 3) Other

22. What medications do you think you need to be aware of when prescribing? Please write drugs and side effects together.

On the recognition and experience of genetic testing

23. Have you ever prescribed a genetic test?

    1) Yes 2) No

24. If you have a prescribed experience, what is the purpose?

    1) Cancer target therapy
    2) Rare disease diagnosis
    3) Chronic disease risk prediction
    4) Prenatal screening
    5) Pharmacogenomics
    6) Do not prescribe

25. Where did you get the knowledge and information about the genetic screening prescription?

    1) Pubmed/Journals
    2) Workshop
    3) Through undergraduate/graduate education
    4) Genetics labs
    5) From senior/colleagues
    6) Pharmaceutical company
    7) Pharmacopoeia, drug information books, websites

26. How do you think the genetic variation contributes to each of the representative fields to which genetic testing is applied?

	Not Important	Less important	Important	Fairly important	Very important
Cancer target therapy					
Rare disease diagnosis					
Chronic disease risk prediction					
Prenatal screening					
Pharmacogenomics					

On the recognition and experience of drug genome screening

27. Have you ever prescribed a pharmacogenomics test?

    1) Yes 2) No

28. If you have prescribed a pharmacogenomics test to your patient, what is its purpose?

    1) Cancer target therapy
    2) Rare disease diagnosis
    3) Chronic disease risk prediction
    4) Prenatal screening
    5) Pharmacogenomics
    6) Increase clinical trial efficiency

29. To what extent do you consider the use of pharmacogenomics test in future clinical studies and research?

	1	2	3	4	5	
Not at all						Actively considering

30. If you plan to use the pharmacogenomics test for medical practice and research, at what point do you expect it to be available?

1	2	3	4	5
Within a year	1-2 years	2-3 years	3-5 years	5 years or later

31. What are the biggest obstacles to the use of pharmacogenomics in clinical practice and research?

1) Low number of patients who benefit from testing
2) Insufficient academic background to use for medical treatment
3) Insufficient genetic knowledge of prescribing physicians
4) Lack of institutional support such as regulation and insurance
5) Inadequate infrastructure for genome inspection of medical institutions
6) It is difficult to explain to the patient the validity of the test
7) Other

Pharmacogenomic testing pattern and cost

32. The FDA recommends taking into consideration the genetic variation information of over 150 drugs whose clinical relevance is related to drug and genetic mutations. The following drugs represent the relationship between some drugs and side effects. If I prescribe a drug-by-drug genetic test to prevent the side effects of the following pairs, what is the most appropriate cost for the patient's own cost?

Drug-side effect pairs	<10$	10 to 50$	50 to 100$	100 to 300$	>300$
Warfarin-bleeding					
Carbamazepine-SJS/TEN					
Simvastatin-Myopathy					
Clopidogrel-MI, Death, stroke					
Valproic acid-Hyperammonia					

33. Pharmacogenomic testing of this study provides personal risk prediction results by

calculating individual genetic variation in drug-related genes of all drugs using data obtained from whole exome sequencing. If you apply this test to a patient, what do you think is the appropriate cost for the patient's out of pocket payment?

1) <50$

2) 50~100$

3) 100~300$

4) 300~500$

5) 500~1,000$

6) >1,000$

7) Other

34. What is the ATC class of the drug you are most interested in predicting drug side effects through pharmacogenomics analysis?

Check all that apply.

A: Digestive tract and metabolism

B: Blood and hematopoietic organ

C: cardiovascular

D: Dermatology

G: Genitourinary and hormonal systems

H: Systemic hormone preparations other than sex hormones and insulin

J: Systemic antiinfectives

L: antitumor agent and immunomodulator

M: Musculoskeletal system

N: Nervous system

P: Insecticides, insecticides and repellents

R: Respiratory system

S: Sensory organs

V: Other

35. If you find out that the risk of a particular drug is high through a drug-genomic test, would you change the prescription as an alternative drug?Mark only one oval.

1) Yes, I will change my prescription.

2) No. I will not change my prescription.

3) I do not know.

4) Other:

36. Would you advise the family, not the patient, to use a genomic analysis to predict drug side effects?

1) Yes, I will recommend it.

2) No, I will not recommend it.

- End of the pre-survey

<This section below contains questions added only in post survey.>

Genome Analysis Drug Risk Assessment Report Evaluation

The following items are used to check your pharmacogenomics report and your application experience. If you answer it freely, it will help you improve and utilize the report in the future.

1. What was the convenience of using the pharmacogenomics report?

	1	2	3	4	5	
Very inconvenience						Very easy to use

2. Why was it difficult to use the report?

1) Term not familiar

2) Inappropriate drug selection

3) Insufficient explanation

4) Not familiar with genes

5) Drug score is not understood

6) Appropriate placement of pictures or graphs

3. How credible is the report?

	1	2	3	4	5	
Reliability is very low						Reliability is very low

4. If you thought the report was unreliable, why?

5. How useful do you think the report is?

6. Have you ever had any side effects of medications that you predicted to be dangerous in this report?

1) Yes 2) No

7. Please write any medicines you would like to add to the report.

8. Please write any medicines you would like to remove to the report.
