# Supplementary material for: Personal genome testing on physicians improves attitudes on pharmacogenomic approaches
Source: PLoS One. 2019 Mar 28;14(3):e0213860. doi: 10.1371/journal.pone.0213860 (PMC6438681; doi:10.1371/journal.pone.0213860)
Supplement: S2 File — (PDF) [file pone.0213860.s005.pdf]

DoctorSeq Project: 사전설문지

본 설문지는 의사들을 대상으로 약물관련 유전체 검사 전후의 인식 변화를 확인하고자 설계되었습니다. 선생님께서 동의서와 함께 제출하신 혈액은 엑솜시퀀싱 (Whole exome sequencing)을 수행하였으며, 이 데이터를 이용하여 약물 관련 유전변이 분석을 진행하였습니다.
본 설문은 약물 관련 유전변이 분석 보고서를 드리기 전에 시행하는 1차 설문이며, 이 설문을 완료하시면 선생님께 선생님의 약물 관련 유전자의 변이 분석을 통한 약물 안전성 예측 결과 보고서를 제공해드리게 됩니다. 보고서를 받으신 후 약 2주 후에 다시 2차 설문 연구가 진행되어 보고서를 받아 보신 전 후의 인식 변화를 확인하게 됩니다.
본 설문은 총 35문항으로 소요시간은 약 20분입니다. 선생님께서 응답하신 내용은 모두 익명으로 처리됩니다.

* Required

사전-사후 설문 비교를 위한 ID 매칭 절차

1. **사전-사후 설문 결과의 변화를 추적하기 위해 ID 매칭 작업이 필요합니다. 핸드폰 번호를 기입해 주십시오. 이 번호는 설문지 매칭 작업 이외에는 사용되지 않습니다.** *

____

기본정보

2. **당신의 성별은 무엇입니까?** *
Mark only one oval.
- ◯ 남성
- ◯ 여성

3. **당신의 나이는 어느 범위에 있습니까?** *
Mark only one oval.
- ◯ 20대
- ◯ 30-39세
- ◯ 40-49세
- ◯ 50-59세
- ◯ 60세 이상

4. **당신의 수련정도는 어느 단계입니까?**
최종 수련단계에 가까운 한개의 타원형에만 표시하십시오.
Mark only one oval.

- 의과대학 졸업
- 인턴수료
- 전문의
- 임상강사 혹은 펠로우쉽 이상
- 부교수 이상
- Other: ____________________

5. **당신의 현재 근무지는 어디입니까?**
Mark only one oval.

- 대학 혹은 의과대학
- 3차병원 혹은 대학병원
- 2차병원 (30-99병상)
- 의원 혹은 개인병원
- 연구소
- 회사
- Other: ____________________

6. **당신의 현재 근무지역은 어디입니까?**
Mark only one oval.

- 서울
- 경기도
- 강원도
- 충청도
- 경상도
- 전라도
- 제주도
- Other: ____________________

7. **당신의 전문과목은 무엇입니까?**

Mark only one oval.

- 가정의학과
- 내과
- 마취통증의학과
- 방사선종양학과
- 병리과
- 비뇨기과
- 산부인과
- 산업의학과
- 신경과
- 신경외과
- 성형외과
- 소아청소년과
- 안과
- 이비인후과
- 예방의학과
- 응급의학과
- 영상의학과
- 외과
- 정신건강의학과
- 정형외과
- 재활의학과
- 진단검사의학과
- 피부과
- 흉부외과
- 핵의학과
- Other: ______________

일반적 건강 상태 조사

8. **현재 앓고 계시거나 이전에 진단받은 적이 있는 질환에 모두 표시해주십시오.**
Check all that apply.

- [ ] 뇌졸중
- [ ] 협심증 등 심장질환
- [ ] 고혈압
- [ ] 고지혈증
- [ ] 당뇨병
- [ ] 폐결핵
- [ ] 만성 기관지염 등 폐질환
- [ ] 암
- [ ] 자가면역성질환
- [ ] 알러지
- [ ] 지방간, 간염, 간경화 등 간질환
- [ ] 갑상성관련 질환
- [ ] 골다공증 등 뼈 관련 질환
- [ ] 없음
- [ ] Other: ____________________

9. **전신 마취를 동반한 수술을 받으신 적이 있습니까?**
Mark only one oval.

- ( ) 예
- ( ) 아니오

약물 부작용 경험에 관한 조사

10. **현재 매일 1회 이상, 3개월 이상 꾸준히 복용해오신 약이 있습니까?**
Mark only one oval.

- ( ) 예, 있습니다.
- ( ) 아니오, 없습니다.

11. **3개월 이상 꾸준히 복용하는 처방약이 있으시다면, 해당 약을 복용하면서 부작용을 경험한 적이 있습니까?**
Mark only one oval.

- ( ) 예, 부작용을 경험한 적이 있습니다.
- ( ) 아니오, 부작용을 경험한 적이 없습니다.
- ( ) 꾸준히 복용하는 약이 없습니다.

12. **지난 일주일동안 한번이라도 약을 복용하신 적이 있습니까? (처방약과 일반약 모두 포함)**
Mark only one oval.

- ( ) 예, 있습니다.
- ( ) 아니오, 없습니다.

13. **지난 일주일동안 한번이라도 약을 복용하셨다면, 그 약의 이름을 기억나는대로 써주십시오.**

14. **현재까지 한번이라도 약물 부작용을 경험하신 적이 있습니까?**
Mark only one oval.

- 예, 약물 부작용을 경험한 적이 있습니다.
- 아니오, 약물 부작용을 경험한 적이 없습니다.
- 약을 먹어본 적이 없습니다.
- Other:

15. **한번이라도 약물 부작용을 경험하셨다면, 그 부작용 정도는 어느 정도 였습니까?**
Mark only one oval.

	1	2	3	4	5	
일시적이며 가벼운 부작용						매우 심각해서 즉각적으로 약을 바꿔야 하는 부작용

16. **약물 부작용을 경험한 적이 있다면, 그 약물과 부작용을 생각나시는대로 써 주십시오. (예, 부루펜-속쓰림, augmentin-urticaria..)**

처방 약물의 예상 부작용에 관한 설문조사

17. **환자가 처음 복용하는 약물의 경우, 약물 부작용 발생 가능성을 환자에게 어느 정도 자주 설명하십니까?**
Mark only one oval.

	1	2	3	4	
거의 설명하지 않는다.					항상 설명한다.

18. **위의 답변에서 약물 부작용 발생 가능성을 설명하지 않는 경우, 그 주된 이유는 무엇입니까?**
Check all that apply.

- [ ] 예측되는 약물 부작용이 경미하기 때문
- [ ] 약물 부작용 발생 가능성이 매우 낮기 때문
- [ ] 약물 부작용 예측이 불가능하기 때문
- [ ] 약물 부작용이 발생하더라도 대체 약물이 없기 때문
- [ ] 환자이 약물 순응도를 떨어뜨릴 수 있기 때문
- [ ] 진료시간이 촉박해서 설명할 시간이 부족하기 때문
- [ ] Other: ____________________

19. **소화불량이나 가려움증 등의 경미한 경우부터 입원 및 사망에 이르는 심각한 경우까지, 다양한 수준의 약물 부작용을 고려했을때, 선생님께서 처방하셨던 환자 중, 얼마나 많은 환자가 약물 부작용을 경험했을 것이라고 생각하십니까?**
(환자가 보고한 경우만 포함하지마시고, 예측한 수준을 표시해주십시오.)
Mark only one oval.

- ( ) 5% 미만
- ( ) 5~10%
- ( ) 10~20%
- ( ) 20~30%
- ( ) 30~50%
- ( ) 50% 이상
- ( ) Other: ____________________

20. **약물 부작용 중 부작용으로 입원을 고려해야 하거나 장애 혹은 사망을 일으키는 심각한 부작용 (Severe ADR) 만 고려했을때, 선생님께서 처방하셨던 환자 중, 얼마나 많은 환자가 심각한 약물 부작용을 경험했을 것이라고 생각하십니까?**
Mark only one oval.

- ( ) 0.1% 미만
- ( ) 0.1~0.5%
- ( ) 0.5~1%
- ( ) 1~5%
- ( ) 5~10%
- ( ) 10~20%
- ( ) 20~30%
- ( ) 30% 이상
- ( ) Other: ____________________

21. **약물 부작용에 관여하는 여러가지 요인들 (나이, 성별, 키/체중, 질병상태, 유전적 소인, 약물 상호작용 등) 중에서, 유전적 소인으로 인한 경우가 약물 부작용에서 차지하는 비중은 어느 정도로 예상하십니까?**

Mark only one oval.

- 5% 미만
- 5~10%
- 10~20%
- 20~30%
- 30~50%
- 50~70%
- 70% 이상
- Other: ______________

22. **약물 부작용에 관여하는 여러가지 요인들 (나이, 성별, 키/체중, 질병상태, 유전적 소인, 약물 상호작용 등) 중에서, 유전적 소인으로 인한 경우가 약물 부작용에서 차지하는 비중은 어느 정도로 예상하십니까? 이때 약물 부작용은 매우 심각한 경우 (입원, 장애, 사망을 초래하는)에 한정해서 응답해주십시오.**

Mark only one oval.

- 5% 미만
- 5~10%
- 10~20%
- 20~30%
- 30~50%
- 50~70%
- 70% 이상
- Other: ______________

23. **환자에게 약을 처방할 때, 특별히 약물 부작용에 대해 주의할 필요가 있다고 생각하는 약물이 있습니까?**

Mark only one oval.

- 예, 있습니다.
- 아니오, 없습니다.
- Other: ______________

24. **처방시 주의할 필요가 있다고 생각하시는 약물은 무엇입니까? 약물과 부작용을 같이 써 주십시오. (약물-부작용)**

____

____

____

____

____

25. **선생님께서 처방했던 약물 중, 환자들이 가장 부작용을 많이 호소하는 약물은 무엇입니까? (환자들이 가장 많이 부작용을 보고했던 약물) 약물과 부작용을 같이 써 주십시오. (예: 아스피린-멍)**

____

26. **선생님께서 처방했던 약물 중, 환자들이 두번째로 부작용을 많이 호소했던 약물은 무엇입니까? 약물과 부작용을 같이 써 주십시오.**

____

27. **선생님께서 처방했던 약물 중, 환자들이 세번째로 부작용을 많이 호소했던 약물은 무엇입니까? 약물과 부작용을 같이 써 주십시오.**

____

유전체 검사에 대한 인식 및 경험에 관하여

28. **선생님께서는 환자에게 직접 유전체 검사를 처방해보신적이 있습니까?**
Mark only one oval.

- ( ) 예, 처방해보았습니다.
- ( ) 아니오, 아직까지 처방하지 않았습니다.

29. **유전체 검사를 처방해보셨던 경우에 어떤 용도로 활용하기 위해 처방하셨습니까?**
Check all that apply.

- [ ] 암유전체 검사
- [ ] 희귀 질환 진단 및 치료
- [ ] 산전진단
- [ ] 질병위험도 예측 및 건강관리
- [ ] 약물 유전체
- [ ] 처방해보지 않음
- [ ] Other: ________________

30. **유전체 검사를 처방하셨을때, 검사에 대한 정보는 어떤 경로로 얻으셨습니까?**
Check all that apply.

- [ ] 학부/대학원 교육
- [ ] 학회 또는 보수교육
- [ ] 선배 혹은 동료의사
- [ ] 제약회사
- [ ] 진단검사 수탁기관
- [ ] 약전 혹은 약품정보서적 및 웹사이트
- [ ] Pubmed 등을 통한 논문 정보
- [ ] 처방해보지 않음
- [ ] Other: ________________

31. **선생님께서 생각하시는 유전체 검사의 목적별 중요도를 알아보고자 합니다. 다음 각 분야에서 유전체 검사의 중요성은 어느 정도 된다고 생각하십니까?**
Mark only one oval per row.

	전혀 중요하지 않다.	약간 중요하다.	보통이다.	상당히 중요하다.	매우 중요하다.
암의 진단 및 항암치료	◯	◯	◯	◯	◯
희귀질환 진단 및 치료	◯	◯	◯	◯	◯
산전진단	◯	◯	◯	◯	◯
질병위험도 예측 및 건강관리	◯	◯	◯	◯	◯
맞춤형 약물 치료	◯	◯	◯	◯	◯

약물 유전체 검사에 대한 인식 및 경험에 관하여

32. **약물 유전체 검사 결과를 진료에 활용하기 위해 환자에게 처방하신 경험이 있습니까? (암 맞춤요법의 경우 포함)**
Mark only one oval.

◯ 예, 있습니다.
◯ 아니오, 없습니다.

33. **약물 유전체 검사를 환자에게 처방하신 적이 있다면, 그 세부 목적은 무엇이었습니까?**
Check all that apply.

☐ 항암제 맞춤요법
☐ 신약 후보 물질 발굴
☐ 약물 적정 용량 예측
☐ 약물 유해반응 예방
☐ 임상시험 효율 증대
☐ 처방경험 없슴
☐ Other: ______

34. **선생님께서는 추후 약물 유전체 검사를 진료 및 연구에 활용할 것을 어느 정도나 고려하고 계십니까?**
Mark only one oval.

	1	2	3	4	5	
전혀 고려하고 있지 않다.	◯	◯	◯	◯	◯	적극적으로 고려하고 있다.

35. **만약 약물 유전체 검사를 진료 및 연구에 활용할 계획이시라면, 어느 정도 시점에서 가능할 것으로 예상하십니까?**
Mark only one oval.

◯ 1년 이내
◯ 1~2년 사이
◯ 2~3년 사이
◯ 3~5년 사이
◯ 5년 이후

36. **약물 유전체 검사를 진료 및 연구에 활용하기에 앞서 가장 큰 장애물이라고 생각되는 것을 3가지만 선택해주십시오.**
Check all that apply.

- [ ] 검사로부터 이득을 얻는 환자수가 적음
- [ ] 진료에 사용할 학술적 근거가 부족함
- [ ] 처방하는 의료인들의 유전체 지식이 충분하지 않음
- [ ] 규제 및 보험 수가 등 제도적 뒷받침이 부족
- [ ] 의료기관의 유전체 검사를 위한 인프라 구축 미비
- [ ] 환자에게 검사의 당위성을 설명하기가 어려움
- [ ] Other: ______

약물 유전체 검사 처방 행태와 비용에 관하여

37. **미국 FDA에서는 약물과 유전변이의 관계에 대해 임상적 근거가 확립된 약물 150여개에 대해, 해당 약물 처방시 유전변이 정보를 고려하도록 권고하고 있습니다. 아래 약물은 그 중 일부 약과 부작용의 관계를 나타냅니다. 아래 쌍에 대해 해당 부작용을 예방하기 위해 약물별 유전체 검사를 처방한다고 할때, 환자 본인 부담 비용으로 가장 적당하다고 생각하는 비용은 어느 정도 입니까?**
Mark only one oval per row.

	1만원 이하	1~5만원	5~10만원	10~30만원	30만원 이상
Warfarin-Bleeding	◯	◯	◯	◯	◯
Carbamazepine-SJS/TEN	◯	◯	◯	◯	◯
Simvastatin-Myopathy	◯	◯	◯	◯	◯
Clopidogrel-Myocardiac Infarction, Death, Stroke	◯	◯	◯	◯	◯
Valproic acid- Hyperammonia	◯	◯	◯	◯	◯

38. **본 연구의 약물 유전체 분석은 전장 엑솜 시퀀싱을 통해 얻어진 데이터를 활용하여 모든 약물의 약물 관련 유전자에 발생한 개인 유전 변이 계산을 통한 개인별 위험도 예측 결과를 제공합니다. 이 검사를 환자에게 적용한다면, 환자 본인 부담 비용은 어느 정도가 적절할 것으로 생각하십니까? (엑솜시퀀싱 비용 포함)**
Mark only one oval.

- ◯ 5만원 이하
- ◯ 5~10만원
- ◯ 10~30만원
- ◯ 30~50만원
- ◯ 50~100만원
- ◯ 100만원 이상
- ◯ Other: ______

39. **약물 유전체 분석을 통한 약물 부작용 예측에서 가장 관심있게 보시는 약물(군) 의 ATC 클래스는 어떤 것입니까?**
Check all that apply.

- [ ] A: 소화관 및 대사
- [ ] B: 혈액 및 조혈기관
- [ ] C: 심혈관계
- [ ] D: 피부과
- [ ] G: 비뇨 생식기계 및 호르몬
- [ ] H: 성호르몬 및 인슐린을 제외한 전신성 호르몬 제제
- [ ] J: 전신작용 항감염제
- [ ] L: 항종양제 및 면역 조절제
- [ ] M: 근골격계
- [ ] N: 신경계
- [ ] P: 구충제, 살충제 및 기피제
- [ ] R: 호흡기계
- [ ] S: 감각기관
- [ ] V:기타

40. **선생님께서는 약물 유전체 검사를 통해 특정 약물의 위험도가 높다는 것을 알게 되신다면, 그 대체 약물로 처방을 변경하시겠습니까?**
Mark only one oval.

- ( ) 예, 처방을 변경하겠습니다.
- ( ) 아니오. 처방을 변경하지 않겠습니다.
- ( ) 모르겠습니다.
- ( ) Other: ____________________

41. **선생님께서는 환자가 아닌 가족에게 유전체 분석을 이용한 약물 부작용 예측 검사를 권고하시겠습니까?**
Mark only one oval.

- ( ) 예, 권고하겠습니다.
- ( ) 아니오, 권고하지 않겠습니다.

42. **위의 질문에서 가족에게 유전체 분석을 통한 약물 부작용 예측 검사를 권고하지 않으시겠다고 답하신 경우, 그 이유는 무엇입니까?**

____
____
____
____
____

설문에 응답해주셔서 감사합니다.

Powered by
Google Forms
