# Supplementary material for: Personal genome testing on physicians improves attitudes on pharmacogenomic approaches
Source: PLoS One. 2019 Mar 28;14(3):e0213860. doi: 10.1371/journal.pone.0213860 (PMC6438681; doi:10.1371/journal.pone.0213860)
Supplement: S3 File — (PDF) [file pone.0213860.s006.pdf]

약물유전체 검사와 약물유해반응에 대한 인식 변화 연구 (2차 설문)

안녕하십니까?

본 설문지는 의사들의 약물관련 유전체검사 후 나타나는 인식변화 (주로 약물부작용 및 약물유전체학을 중심으로)에 대한 연구를 위해 설계되었습니다. 선생님께서는 이미 1차 설문을 완료하시고 약물 관련 유전자 분석 결과 보고서를 받아보셨을 것입니다. 본 설문지는 그 이후 인식 변화를 확인하기 위해 설계된 2차 설문입니다. 본 설문지를 통해 선생님께서 응답하신 내용은 익명으로 처리되며, 소요시간은 10분 이내로 예상됩니다.
연구에 참여해주셔서 대단히 감사합니다.

서울대학교 의과대학 정보의학실 연구원 일동

* Required

1. **검사 전후의 설문 결과 변화를 확인하기 위해 ID 매칭 작업이 필요합니다. 1차 설문에서 기입하셨던 핸드폰 번호를 써주시기 바랍니다.** *

____

기본 정보

6문항

2. **당신의 성별은 무엇입니까?** *
Mark only one oval.
- ( ) 남성
- ( ) 여성

3. **당신의 나이는 어느 범위에 있습니까?** *
Mark only one oval.
- ( ) 30대 이하
- ( ) 30~39세
- ( ) 40~49세
- ( ) 50~59세
- ( ) 60세 이상

4. **당신의 수련정도는 어느 단계입니까?** *
Mark only one oval.
- ( ) 의과대학 졸업
- ( ) 인턴수료
- ( ) 전문의
- ( ) 임상강사 혹은 펠로우쉽 이상
- ( ) 부교수 이상
- ( ) Other: ______________________________

5. **당신의 현재 근무지는 어디입니까?** *

복수 선택 가능

Check all that apply.

- [ ] 대학 혹은 의과대학
- [ ] 3차 병원 혹은 대학병원
- [ ] 2차 병원 (30-99병상)
- [ ] 의원 혹은 개인병원
- [ ] 연구소
- [ ] 회사

6. **당신의 현재 근무지역은 어디입니까? (시/도)** *

Mark only one oval.

- 서울시
- 인천시
- 대전시
- 대구시
- 울산시
- 광주시
- 부산시
- 경기도
- 강원도
- 충청도
- 경상도
- 전라도
- 제주도 및 기타 섬지역

7. **당신의 전문과목은 무엇입니까?** *

복수 응답 가능

Check all that apply.

- [ ] 내과
- [ ] 외과
- [ ] 산부인과
- [ ] 소아청소년과
- [ ] 정신건강의학과
- [ ] 정형외과
- [ ] 안과
- [ ] 이비인후과
- [ ] 피부과
- [ ] 비뇨기과
- [ ] 영상의학과
- [ ] 신경외과
- [ ] 진단검사의학과
- [ ] 병리과
- [ ] 예방의학과
- [ ] 마취통증의학과
- [ ] 흉부외과
- [ ] 성형외과
- [ ] 신경과
- [ ] 방사선종양학과
- [ ] 재활의학과
- [ ] 가정의학과
- [ ] 산업의학과
- [ ] 핵의학과
- [ ] 응급의학과
- [ ] 전문과목 수련을 하지 않음
- [ ] Other: ____________________

일반적 건강상태 및 약물 복용력 조사

3 문항

8. **현재 앓고 계시거나 이전에 진단받은 적이 있는 질환에 표시해주십시오.**
복수 응답 가능
Check all that apply.

- [ ] 뇌졸중
- [ ] 심장질환
- [ ] 고혈압
- [ ] 당뇨병
- [ ] 폐결핵
- [ ] 만성 기관지염 등 폐질환
- [ ] 암
- [ ] 자가면역성 질환
- [ ] 알러지
- [ ] 지방간, 간염, 간경화 등 간질환
- [ ] 갑상선 관련 질환
- [ ] 골다공증 등 뼈관련 질환
- [ ] 없음
- [ ] Other: ____________

9. **전신 마취를 동반한 수술을 받으신 적이 있습니까?**
Mark only one oval.

- ( ) 예
- ( ) 아니오

10. **현재 매일 1회 이상 3개월 이상 복용해 오신 처방약이 있습니까?**
Mark only one oval.

- ( ) 예, 있습니다. *Skip to question 11.*
- ( ) 아니오, 없습니다. *Skip to question 13.*

장기 복용약 부작용 경험에 관하여

2 문항

11. **항상 복용하는 처방약이 있으시다면, 약을 복용하면서 부작용을 경험하신 적이 있습니까?**
Mark only one oval.

- ( ) 예. 부작용을 경험한 적이 있습니다.
- ( ) 아니오. 부작용을 경험한 적이 없습니다.

12. **항상 복용하시던 약에서 약물 부작용을 경험한 적이 있다면, 그 부작용 정도는 어느 정도였습니까?**
Mark only one oval.

	1	2	3	4	5	
일시적이며 가벼운 부작용	( )	( )	( )	( )	( )	매우 심각해서 즉각적으로 약을 바꿔야 하는 부작용

단기 복용약 부작용 경험에 관하여

13. **매일 복용하는 만성질환 치료제를 제외하고, 지난 일주일 동안 한번이라도 복용한 약물이 있으십니까? (처방약과 일반약을 포함해서)**
Mark only one oval.

- ( ) 예.
- ( ) 아니오.

14. **현재까지 한번이라도 약물 부작용을 경험하신 적이 있습니까?**
Mark only one oval.

- ( ) 예. 약물 부작용을 경험한 적이 있습니다.
- ( ) 아니오. 약물 부작용을 경험한 적이 없습니다.
- ( ) 약을 먹어본 적이 없습니다.
- ( ) Other: ____________

15. **한번이라도 약물 부작용을 경험한 적이 있다면, 그 부작용 정도는 어느 정도였습니까?**
Mark only one oval.

	1	2	3	4	5	
일시적이며 가벼운 부작용	( )	( )	( )	( )	( )	매우 심각해서 즉각적으로 약을 바꿔야 하는 부작용

환자에게 처방하는 약물에 대한 부작용 예상

7문항

16. **환자에게 처음 처방하는 약물의 경우 환자에게 약물 부작용 발생 가능성을 어느 정도 자주 설명하십니까?**
Mark only one oval.

- ( ) 설명하지 않음
- ( ) 때에 따라 설명함
- ( ) 항상 설명함
- ( ) Other: ____________

17. **위의 답변에서 약물 부작용 발생 가능성을 설명하지 않는 경우, 그 주된 이유는 무엇입니까? (중복선택)**
Check all that apply.

- [ ] 예측되는 약물 부작용이 경미하기 때문
- [ ] 약물 부작용 예측이 불가능 하기 때문
- [ ] 약물 부작용이 매우 드물기 때문
- [ ] 약물 부작용이 발생하더라도 대체 약물이 없기 때문
- [ ] 환자의 약물 순응도를 떨어뜨릴 수 있기 때문
- [ ] 진료 시간이 촉박해서 설명할 시간이 부족하기 때문
- [ ] 약물 부작용 발생 가능성을 설명함
- [ ] Other: ____________

18. **소화불량이나 가려움증 등 경미한 경우부터 입원 및 사망 등 심각한 경우까지 다양한 수준의 약물 부작용을 고려했을 때, 선생님께서 약물을 처방하셨던 환자 중, 얼마나 많은 환자가 부작용을 경험했을 것이라고 생각하십니까?**

(환자가 보고한 경우만 포함시키지 마시고, 예측한 수준을 표시해주십시오.)

Mark only one oval.

- 5% 미만
- 5%~10%
- 10%~20%
- 20%~30%
- 30%~50%
- 50% 이상
- Other: ____________

19. **약물 부작용에 관여하는 여러가지 요인들 (나이, 성별, 질병상태, 유전적 소인, 약물 상호작용등) 중에서, 유전적 소인으로 인한 경우가 약물 부작용에서 차지하는 비중은 어느정도로 예상하십니까?**

Mark only one oval.

- 5% 미만
- 5~10%
- 10~20%
- 20~30%
- 30~50%
- 50~70%
- 70% 이상
- Other: ____________

20. **약물 부작용 중 부작용으로 입원을 고려해야 하거나 장애 혹은 사망을 일으키는 심각한 부작용 (Severe ADR) 만 고려했을때, 선생님께서 처방하셨던 환자 중, 얼마나 많은 환자가 심각한 약물 부작용을 경험했을 것이라고 생각하십니까?**

Mark only one oval.

- 0.1% 미만
- 0.1~0.5%
- 0.5~1%
- 1~5%
- 5~10%
- 10~20%
- 20~30%
- 30% 이상
- Other: ____________

21. **약물 부작용에 관여하는 여러가지 요인들 (나이, 성별, 키/체중, 질병상태, 유전적 소인, 약물 상호작용 등) 중에서, 유전적 소인으로 인한 경우가 약물 부작용에서 차지하는 비중은 어느 정도로 예상하십니까? 이때 약물 부작용은 매우 심각한 경우 (입원, 장애, 사망을 초래하는)에 한정해서 응답해주십시오.**

Mark only one oval.

- 5% 미만
- 5~10%
- 10~20%
- 20~30%
- 30~50%
- 50~70%
- 70% 이상
- Other: ____________

약물유전체 검사에 대한 인식 및 경험에 관하여

9문항

22. **선생님께서는 환자에게 직접 유전체 검사를 처방해 보신 적이 있습니까?**

Mark only one oval.

- 네. 처방했습니다.
- 아니오. 아직까지 처방하지 않았습니다.

23. **유전체 검사를 처방해보셨던 경우에 어떤 용도로 활용하기 위해 처방하셨습니까?**

복수응답 가능

Check all that apply.

- 암 유전체검사
- 희귀질환 진단 및 치료
- 산전진단
- 질병 위험도 예측 및 건강관리
- 약물 유전체
- 처방해 보지 않음
- Other: ____________

24. **유전체 검사를 처방하셨을때, 검사에 대한 정보는 어떤 경로로 얻으셨습니까? (복수 응답 가능)**

복수응답 가능

Check all that apply.

- 학부/대학원 교육
- 학회 또는 보수교육
- 선배 혹은 동료의사
- 제약회사
- 진단검사 수탁기관
- 약전 혹은 약품 정보 서적 및 웹사이트
- Pubmed 등을 통한 논문 정보
- 처방해 보지 않음
- Other: ____________

25. **선생님께서 생각하시는 유전체 검사의 역할의 중요도를 알아보고자 합니다. 다음 각 분야에서 유전체 검사의 중요성은 어느정도 된다고 생각하십니까?** *

Mark only one oval per row.

	전혀 중요하지 않다	약간 중요하다	보통이다	어느정도 중요하다	매우 중요하다
암의 진단 및 항암치료	⬭	⬭	⬭	⬭	⬭
희귀질환 진단 및 치료	⬭	⬭	⬭	⬭	⬭
산전진단	⬭	⬭	⬭	⬭	⬭
질병위험도 예측 및 건강관리	⬭	⬭	⬭	⬭	⬭
맞춤형 약물 치료	⬭	⬭	⬭	⬭	⬭

26. **약물 유전체 검사 결과를 진료에 활용하기 위해 환자에게 처방한 경험이 있으십니까? (암 맞춤요법의 경우 포함)**

Mark only one oval.

- ⬭ 네. 있습니다.
- ⬭ 아니오. 없습니다.

27. **약물 유전체 검사 결과를 진료에 활용하기 위해 환자에게 처방한 경험이 있으시다면 그 목적은 무엇이었습니까? (복수 응답 가능)**

Check all that apply.

- ☐ 항암제 맞춤요법
- ☐ 신약후보물질 발굴
- ☐ 약물 적정 용량 예측
- ☐ 약물 유해반응 예방
- ☐ 임상 시험 효율 증대
- ☐ 처방경험 없음
- ☐ Other: ____________________

28. **선생님께서는 추후 약물 유전체 검사를 진료 및 연구에 활용할 것을 어느 정도나 고려하고 계십니까?**

Mark only one oval.

	1	2	3	4	5	
전혀 고려하고 있지 않다.	⬭	⬭	⬭	⬭	⬭	적극적으로 고려하고 있다.

29. **만약 약물 유전체 검사를 진료 및 연구에 활용할 계획이시라면, 어느 정도 시점에서 가능할 것으로 예상하십니까?**

Mark only one oval.

- ⬭ 1년 이내
- ⬭ 1~2년 사이
- ⬭ 2~3년 사이
- ⬭ 3~5년 사이
- ⬭ 5년 이후

30. **약물 유전체 검사를 진료 및 연구에 활용하기에 앞서 가장 큰 장애물이라고 생각되는 것을 3가지만 선택해주십시오.**

Check all that apply.

- [ ] 검사로부터 이득을 얻는 환자수가 적음
- [ ] 진료에 사용할 학술적 근거가 부족함
- [ ] 처방하는 의료인들의 유전체 지식이 충분하지 않음
- [ ] 규제 및 보험 수가 등 제도적 뒷받침이 부족
- [ ] 의료기관의 유전체 검사를 위한 인프라 구축 미비
- [ ] 환자에게 검사의 당위성을 설명하기가 어려움
- [ ] Other: ________________

약물 유전체 처방 행태 변화와 비용

5문항

31. **FDA에서는 약물 유전학의 임상적 근거가 확립된 약물 150여가지에 대해 처방시 약물 유전학 정보를 고려하도록 권고합니다. 아래 약물은 그중 일부 약과 부작용 쌍으로, 이에 대한 부작용을 예방하기 위해 약물별 유전체 검사를 처방한다고 할때, 환자 본인 부담 비용으로 가장 적당하다고 생각하는 비용은 어느 정도 입니까?** *

Mark only one oval per row.

	1만원 이하	1~5만원	5~10만원	10~30만원	30만원 이상
Warfarin-Bleeding	◯	◯	◯	◯	◯
Carbamazepine-SJS/TEN	◯	◯	◯	◯	◯
Simvastatin-Myopathy	◯	◯	◯	◯	◯
Clopidogrel-Myocardiac infarction, Death, Stroke	◯	◯	◯	◯	◯
Valproic acid-Hyperammonemia	◯	◯	◯	◯	◯

32. **본 연구의 약물 유전체 분석은 전체 엑솜 시퀀싱을 통해 얻어진 데이터를 활용하여, 모든 약물에 대해 유전변이 계산을 통한 개인별 위험도 예측 결과를 제공하고 있습니다. 이 검사를 환자에게 적용한다면, 환자 본인 부담 비용은 어느 정도가 적절할 것으로 생각하십니까? (엑솜시퀀싱 비용 포함)**

Mark only one oval.

- ◯ 5만원 이하
- ◯ 5~10만원
- ◯ 10~30만원
- ◯ 30~50만원
- ◯ 50~100만원
- ◯ 100만원 이상
- ◯ Other: ________________

33. **약물 유전체 분석을 통한 약물 부작용 예측에서 가장 관심있게 보시는 약물(군)의 ATC 클래스는 어떤 것입니까?**
Check all that apply.

- [ ] A: 소화관 및 대사
- [ ] B: 혈액 및 조혈기관
- [ ] C: 심혈관계
- [ ] D: 피부과
- [ ] G: 비뇨생식기계 및 성호르몬
- [ ] H: 성호르몬 및 인슐린을 제외한 전신성 호르몬 제제
- [ ] J: 전신작용 항감염제
- [ ] L: 항종양제 및 면역 조절제
- [ ] M: 근골격계
- [ ] N: 신경계
- [ ] P: 구충제, 살충제 및 기피제
- [ ] R: 호흡기계
- [ ] S: 감각기관
- [ ] V: 기타

34. **선생님께서는 약물 유전체 검사를 통해 특정 약물의 위험도가 높다는 것을 알게 되신다면, 그 대체 약물로 처방 변경을 고려하시겠습니까?**
Mark only one oval.

- ( ) 네. 고려하겠습니다.
- ( ) 아니오.고려하지 않겠습니다.
- ( ) 모르겠습니다.

35. **선생님께서는 환자가 아닌 가족에게 유전체 분석을 이용한 약물 부작용 예측 검사를 권고하시겠습니까?**
Mark only one oval.

- ( ) 예. 권고하겠습니다.
- ( ) 아니오. 권고하지 않겠습니다.

유전체 분석 약물 위험성 평가 보고서 평가

아래 항목은 선생님의 개인 유전체 분석을 통한 약물 위험성 평가 보고서와 애플리케이션 사용 경험을 확인하기 위한 항목입니다. 자유롭게 답변해주시면 앞으로 보고서 개선 및 활용에 큰 도움이 되겠습니다.

36. **약물 위험성 평가 보고서를 사용하시는데 편리성은 어느 정도였습니까?**
Mark only one oval.

	1	2	3	4	5	
매우 불편했다.	( )	( )	( )	( )	( )	매우 사용이 용이했다.

37. **약물 위험성 평가 보고서를 이용하시기 어려우신 부분이 있었다면 어떤 점이었습니까?**

복수 응답

Check all that apply.

- [ ] 용어가 익숙하지 않았음
- [ ] 약물 선정이 부적절함
- [ ] 설명이 부족함
- [ ] 유전자에 익숙하지 않음
- [ ] 약물 점수가 이해되지 않음
- [ ] 그림이나 그래프의 배치가 적절하지 않음
- [ ] Other: ______

38. **약물 위험성 평가 보고서의 신뢰도는 어느 정도라고 생각하십니까?**

Mark only one oval.

	1	2	3	4	5	
신뢰도가 매우 낮다.	◯	◯	◯	◯	◯	신뢰도가 매우 높다.

39. **약물 부작용 보고서의 신뢰도가 낮다고 생각하셨다면 그 이유는 무엇이었습니까?**

______

40. **약물 위험성 평가 보고서의 유용성은 어느 정도라고 생각하십니까?**

Mark only one oval.

	1	2	3	4	5	
거의 유용하지 않다.	◯	◯	◯	◯	◯	매우 유용하다.

41. **약물 위험성 평가 보고서에서 위험하다고 예측한 약물 중에 부작용을 경험하신 약이 있었습니까?**

Mark only one oval.

- ◯ 있었다.
- ◯ 없었다.
- ◯ Other: ______

42. **약물 위험성 평가 보고서에서 위험하다고 예측한 약물 중에 부작용을 경험하신 약이 있었다면 어떤 약이었습니까?**

______

43. **약물 위험성 평가 보고서에 추가하고 싶으신 약이 있다면 써주십시오.**

______

44. **약물 위험성 평가 보고서에 제거하고 싶으신 약이 있다면 써주십시오.**

______

설문에 응답해주셔서 감사합니다.

Powered by
Google Forms
